# Arsenic in drinking water: An analysis of global drinking water regulations and recommendations for updates to protect public health

**DOI:** 10.1371/journal.pone.0263505

**Published:** 2022-04-06

**Authors:** Seth H. Frisbie, Erika J. Mitchell

**Affiliations:** 1 Department of Chemistry and Biochemistry, Norwich University, Northfield, Vermont, United States of America; 2 Better Life Laboratories, Inc., East Calais, Vermont, United States of America; Harvard School of Public Health, UNITED STATES

## Abstract

Evidence-based public health policy often comes years or decades after the underlying scientific breakthrough. The World Health Organization’s (WHO’s) provisional 10 μg/L arsenic (As) drinking water guideline was set in 1993 based on “analytical achievability.” In 2011, an additional proviso of “treatment performance” was added; a health-based risk assessment would lead to a lower and more protective guideline. Since the WHO does not require United Nations member states to submit copies of national drinking water regulations, there is no complete database of national drinking water standards or guidelines. In this study, we collated and analyzed all drinking water regulations for As from national governments worldwide. We found regulations for 176 countries. Of these countries, 136 have drinking water regulations that specify 10 μg/L As or less, while 40 have regulations that allow more than 10 μg/L of As; we could not find any evidence of regulations for 19 countries. The number of people living in countries that do not meet the WHO’s guideline constitutes 32% of the global population. Global As regulations are also strongly tied to national income, with high income countries more likely to meet the WHO’s guideline. In this study, we examined the health risk assessments that show a clear need for reducing As exposure to levels far below the current WHO provisional guideline. We also show that advances in analytical chemistry, drinking water treatment, and the possibility of accessing alternative drinking water supplies without As suggest that both low-income countries with limited resources and high-income countries with adequate resources can adopt a lower and more protective national drinking water standards or guidelines for As. Thus, we recommend that regulators and stake holders of all nations reassess the possibilities for improving public health and reducing health care expenses by adopting more stringent regulations for As in drinking water.

## 1. Introduction

Arsenic (As) is a common drinking water contaminant that is often found in groundwater wells [1–6]. Even at very low concentrations, chronic consumption of As in drinking water has been strongly associated with a variety of cancers and other adverse health effects in humans [7–13]. At least 226 million people in 56 countries are exposed to unsafe concentrations of As in drinking water and food [14].

It often takes years or decades for an advance in science to cause a change in public health policy [15]. Notably, we will demonstrate that all national standards and guidelines for As in drinking water are based on outdated assumptions. For example, many low-and middle-income countries still use the World Health Organization’s (WHO’s) 1963 drinking water standard for As of 50 micrograms per liter (μg/L), even though the WHO lowered its recommended maximum concentration for As to 10 μg/L in 1993 [16, 17]. Most high-income countries use a 10 μg/L As drinking water standard, consistent with the current WHO recommended maximum concentration. However, many regulators and stakeholders may not be aware that the WHO 10 μg/L guideline is deemed “provisional” [17, 18]. Although risk assessment data indicate a lower guideline would be more appropriate, the WHO retains the 10 μg/L for As based on an assumed practical quantification limit of routine laboratories, as well as an assumed practical drinking water treatment limit [17, 18]. Much new technology for the testing and treatment of As has been developed since the provisional 10 μg/L WHO guideline was first set based on practical concerns rather than health data, but the provisional guideline value has not been updated since 1993 [17, 18].

This causes a significant threat to global public health. Current research shows that more protective national standards and guidelines for As in drinking water are technologically feasible and urgently necessary to protect public health. In this study we collate all current national standards for As in drinking water into a database that can be used to examine patterns of As regulations based on geographic regions and income. We review the basis of the WHO’s drinking water guideline for As and why it is deemed “provisional”. We examine risk assessment data that indicate that an As guideline of 10 μg/L does not provide sufficient protection against cancer and other adverse health effects. Finally, we review current quantification and treatment technologies, demonstrating the technological feasibility of reducing the maximum allowable concentration of As in drinking water in both high and low income countries.

## 2. Materials and methods

### 2.1 International drinking water standards for arsenic

In order to better understand the state of current regulations for As in drinking water worldwide, we collated all available national standards. We began with a list of the 193 United Nations member states and supplemented this list with 2 other states for which the World Bank provides income group data, Taiwan and Kosovo [19, 20]. We then searched for official laws or decrees promulgated by these national governments regulating As in drinking water. For each country we began our search for national laws and decrees on As in drinking water at the Law Library of Congress website (http://www.loc.gov/law/help/guide/nations.php). When possible, the official online government gazette of a country was also used for this search. If necessary, the FAOLEX database of national laws and regulations on food, agriculture, and renewable natural resources [21], Google Scholar [22], and Google were also used for this search. We also searched catalogs of national standards agencies. When necessary, Google Translate [23] was used to translate between English and the official language of a country. Common search terms included the name of the country, the official gazette of the country, “drinking water quality standards”, “drinking water standards”, “drinking water”, “water”, “arsenic”, “μg/L”, and “mg/L” in both English and the official language of the country. We also compared our search results with those of previous partial surveys of international drinking water regulations [24–27].

For countries in which our search methods could not locate a national law or decree, we continued our search, seeking secondary evidence for regulations such as peer-reviewed articles, dissertations, theses, or similar documents that state a drinking water standard for As in a country. Secondary evidence of regulations was only used when primary evidence was not found. We searched for secondary evidence of regulations using the name of the country, the official gazette of the country, “drinking water quality standards”, “drinking water standards”, “drinking water”, “water”, “arsenic”, “μg/L”, and “mg/L” in both English and the official language of the country. We also used internet searches to determine the national agency or organization responsible for setting drinking water quality standards in these countries and contacted representatives of these agencies via email and Facebook in a national language requesting help obtaining the standards.

If we were unable to find either primary or secondary evidence for a drinking water regulation for As in a country, we assumed that there was most likely no national standard or guideline. There were 19 countries for which we could not find any evidence of drinking water regulations for As.

To understand the relationships between population, income, and As standards, we collated population, gross domestic product (GDP), and GDP per capita data for each country [20, 28, 29]. We selected 2019 population and GDP data since it is the most current data before the demographic and economic upheavals caused by the COVID-19 pandemic. Although COVID-19 was first detected during 2019, as of January, 2020, international economists were not yet expecting it to have a major impact on the world economy [30].

### 2.2 Data analysis and statistics

We used R version 4.1.1, “Kick Things”, released August 10, 2021 to calculate descriptive statistics and perform hypothesis testing. For hypothesis testing, we assumed a 95% confidence level for significance. Multiple statistical comparisons of the data were not made, so no corrections for multiple comparisons were applied. Figures and maps were also created with R using the R packages ggplot2 and ggmaps.

## 3. Results and discussion

The results of our search for national As regulations are listed in Table 1. In addition to the name of each country and concentration of As specified in the regulation, we have also included the 2019 Gross Domestic Product (GDP) per capita from the World Bank [20], the 2019 WB income category [31], whether the stated standard is determined independently by the country or tied to international regulations or guidelines such as the WHO drinking water guideline, and when the regulation was most recently updated.

**Table 1:** International drinking water standards for arsenic and the 2019 gross domestic product (GDP) per capita in United States dollars for 195 countries.

Country	As standard (μg/L)	Year of publication	GDP / Capita (2019) [20]	WB Income Class (2019) [31]	Regulatory Link	Regulation type^a^
**Africa**						
Algeria ^[^^32^^]^	10	2011	$3,974	LM		Law/Decree
Angola ^[^^33^^]^	50	2011	$2,791	LM		Law/Decree
Benin ^[^^34^^]^	50	2001	$1,219	LM		Law/Decree
Botswana ^[^^35^^–^^37^^]^^b^	10	2009	$7,961	UM		Standards Org.
Burkina Faso ^[^^38^^]^	10	2005	$787	Low	WHO: 1996	Law/Decree
Burundi ^[^^39^^,^ ^40^^]^	10	2000	$261	Low	EAS: 2000	Standards Org.
Cameroon ^[^^18^^,^ ^41^^]^	10	2007	$1,507	LM	WHO	Gov. Org.
Cape Verde ^[^^42^^]^	10	2004	$3,604	LM		Law/Decree
Central African Republic ^[^^18^^,^ ^43^^]^	10	2017	$468	Low	WHO	Law/Decree
Chad ^[^^44^^]^	10	2010	$710	Low		Law/Decree
Comoros ^[^^45^^]^	50	1994	$1,370	LM	WHO	Law/Decree
Congo	NA		$2,280	LM		
Democratic Republic of the Congo	NA		$581	Low		
Djibouti ^[^^46^^]^	50	2001	$3,415	LM		Law/Decree
Egypt ^[^^47^^,^ ^48^^]^	10	2007	$3,019	LM		Gov. Org.
Equatorial Guinea	NA		$8,132	UM		
Eritrea	NA		NA	Low		
Ethiopia ^[^^49^^]^	10	2013	$856	Low		Standards Org.
Gabon ^[^^50^^]^	50	2011	$7,767	UM		Gov. Org.
Ghana ^[^^51^^–^^53^^]^	10	2017	$2,202	LM		Standards Org.
Guinea ^[^^54^^]^	10	1997	$963	Low		Law/Decree
Guinea-Bissau	NA		$697	Low		
Ivory Coast ^[^^18^^,^ ^55^^]^	10	2017	$2,276	LM	WHO	Law/Decree
Kenya ^[^^56^^–^^58^^]^	10	2018	$1,817	LM		Standards Org.
Lesotho ^[^^59^^]^	NA		$1,118	LM		
Liberia ^[^^18^^,^ ^60^^]^	10	2017	$622	Low	WHO	Gov. Org.
Libya ^[^^61^^–^^63^^]^	10	2015	$7,686	UM		Standards Org.
Madagascar ^[^^64^^]^	50	2004	$523	Low		Law/Decree
Malawi ^[^^65^^–^^67^^]^	50	2013	$412	Low		Standards Org.
Mali ^[^^68^^]^	10	2007	$879	Low		Gov. Org.
Mauritania ^[^^69^^]^	10	2015	$1,679	LM	WHO	Gov. Org.
Mauritius ^[^^70^^]^	10	1996	$11,099	High		Law/Decree
Morocco ^[^^71^^–^^73^^]^	10	2006	$3,282	LM		Standards Org.
Mozambique ^[^^74^^]^	10	2004	$504	Low		Law/Decree
Namibia ^[^^75^^,^ ^76^^]^	300	1988	$4,957	UM		Law/Decree
Niger ^[^^18^^,^^77^^]^	10	2017	$554	Low	WHO	Law/Decree
Nigeria ^[^^78^^]^	10	2015	$2,230	LM		Standards Org.
Rwanda ^[^^79^^,^ ^80^^]^	10	2014	$820	Low		Standards Org.
São Tomé and Príncipe ^[^^81^^]^	NA		$1,947	LM		
Senegal ^[^^18^^,^ ^82^^]^	10	1996	$1,447	LM		Gov. Org.
Seychelles ^[^^83^^]^	10	2012	$17,448	High		Law/Decree
Sierra Leone ^[^^84^^]^	NA		$528	Low		
Somalia	NA		NA	Low		
South Africa ^[^^85^^,^ ^86^^]^	10	2015	$6,001	UM		Law/Decree
South Sudan ^[^^87^^]^	50	2011		Low		Gov. Org.
Sudan ^[^^88^^,^ ^89^^]^	7	2009	$713	Low		Standards Org.
Swaziland ^[^^85^^,^ ^86^^,^ ^90^^,^ ^91^^]^	10	2015	$4,090	LM		Standards Org.
Tanzania ^[^^92^^–^^94^^]^	10	2018	$1,089	LM	EAS	Standards Org.
The Gambia ^[^^95^^]^	50	2008	$778	Low		Gov. Org.
Togo ^[^^96^^]^	10	2015	$679	Low		Gov. Org.
Tunisia ^[^^97^^]^	10	2013	$3,317	LM		Standards Org.
Uganda ^[^^98^^]^	10	2014	$794	Low	EAS	Standards Org.
Zambia ^[^^99^^]^	10	2010	$1,305	LM		Standards Org.
Zimbabwe ^[^^100^^–^^104^^]^	10	2014	$1,464	LM		Standards Org.
**The Americas**						
Antigua and Barbuda ^[^^105^^]^^c^	10	2003	$17,113	High	CARICOM	Law/Decree
Argentina ^[^^107^^]^	10	2019	$9,912	UM		Law/Decree
Bahamas ^[^^108^^]^^c^	50	2010	$34,864	High	CARICOM	Standards Org.
Barbados ^[^^109^^]^^c^	10	2017	$18,148	High	CARICOM	Gov. Org.
Belize ^[^^110^^]^^c^	NA		$4,815	UM	CARICOM	
Bolivia ^[^^111^^]^	10	2018	$3,552	LM		Gov. Org.
Brazil ^[^^112^^]^	10	2021	$8,655	UM		Law/Decree
Canada ^[^^113^^]^	10	2020	$46,195	High		Gov. Org.
Chile ^[^^114^^]^	10	2007	$14,896	High		Law/Decree
Colombia ^[^^115^^]^	10	2007	$6,429	UM		Gov. Org.
Costa Rica ^[^^116^^]^	10	2015	$12,244	UM		Law/Decree
Cuba ^[^^117^^]^	50	2017	NA	UM		Standards Org.
Dominica ^[^^18^^,^ ^118^^]^^c^	10	2017	$8,111	UM	CARICOM	Gov. Org.
Dominican Republic ^[^^119^^]^	50	2001	$8,282	UM		Gov. Org.
Ecuador ^[^^120^^]^	100	2015	$6,184	UM		Law/Decree
El Salvador ^[^^121^^]^	10	2009	$4,187	LM		Standards Org.
Grenada ^[^^122^^]^^c^^,^^d^	1	2005	$10,809	UM	CARICOM	Law/Decree
Guatemala ^[^^124^^]^	10	2013	$4,620	UM		Standards Org.
Guyana^c^	NA		$6,610	UM	CARICOM	
Haiti ^[^^125^^]^^c^	10	2017	$1,272	Low	WHO	Gov. Org.
Honduras ^[^^126^^]^	10	2007	$2,575	LM		Law/Decree
Jamaica ^[^^127^^]^^c^	NA		$5,582	UM	CARICOM	
Mexico ^[^^128^^]^^e^	10	2019	$10,069	UM		Law/Decree
Nicaragua ^[^^129^^]^	50	2000	$1,913	LM		Law/Decree
Panama ^[^^130^^]^^f^	10	2007	$15,731	High		Law/Decree
Paraguay ^[^^131^^]^^g^	500	2000	$5,415	UM		Law/Decree
Peru ^[^^133^^]^	10	2017	$6,978	UM		Law/Decree
Saint Kitts and Nevis^c^	NA		$19,939	High	CARICOM	
Saint Lucia^c^	NA		$11,611	UM	CARICOM	
Saint Vincent and the Grenadines^c^	NA		$7,458	UM	CARICOM	
Suriname ^[^^134^^]^^c^	NA		$6,360	UM	CARICOM	
Trinidad and Tobago^c^	NA		$17,398	High	CARICOM	
United States ^[^^135^^]^	10	2018	$65,298	High		Gov. Org.
Uruguay ^[^^136^^]^	20	2010	$16,190	High		Standards Org.
Venezuela ^[^^137^^]^	10	1998	NA	UM		Law/Decree
**Asia**						
Afghanistan ^[^^138^^]^	50	2013	$507	Low		Standards Org.
Armenia ^[^^139^^–^^141^^]^	50	2005	$4,623	UM		Law/Decree
Azerbaijan ^[^^142^^,^ ^143^^]^	50	1985	$4,794	UM	CIS	Standards Org.
Bahrain ^[^^144^^,^ ^145^^]^	10	2012	$23,504	High	GCC	Standards Org.
Bangladesh ^[^^146^^]^	50	2019	$1,856	LM		Gov. Org.
Bhutan ^[^^147^^]^	10	2018	$3,316	LM		Gov. Org.
Brunei ^[^^17^^,^ ^148^^]^	10	1993	$31,087	High	WHO: 1993	Gov. Org.
Cambodia ^[^^149^^]^	50	2004	$1,643	LM		Gov. Org.
China ^[^^150^^]^	50	2006	$10,217	UM		Standards Org.
Georgia ^[^^151^^]^	10	2014	$4,698	UM		Law/Decree
India ^[^^152^^]^^h^	10	2012	$2,100	LM		Standards Org.
Indonesia ^[^^153^^]^	10	2010	$4,142	UM		Gov. Org.
Iran ^[^^154^^]^	10	2010	NA	UM		Standards Org.
Iraq ^[^^155^^,^ ^156^^]^	10	2009	$5,955	UM		Standards Org.
Israel ^[^^157^^]^	10	2016	$43,592	High		Law/Decree
Japan ^[^^158^^]^	10	2015	$40,247	High		Gov. Org.
Jordan ^[^^159^^]^	10	2015	$4,405	UM		Standards Org.
Kazakhstan ^[^^160^^]^	50	2015	$9,812	UM		Law/Decree
Kuwait ^[^^145^^,^ ^161^^]^	10	2011	$32,000	High	GCC	Gov. Org.
Kyrgyzstan ^[^^162^^]^	50	2004	$1,309	LM		Law/Decree
Laos ^[^^163^^]^	50	2009	$2,535	LM		Gov. Org.
Lebanon ^[^^164^^]^	50	1999	$7,584	UM		Standards Org.
Malaysia ^[^^165^^]^	10	2004	$11,414	UM		Gov. Org.
Maldives ^[^^166^^]^	10	2017	$10,627	UM		Gov. Org.
Mongolia ^[^^167^^–^^169^^]^	10	2018	$4,340	LM		Standards Org.
Myanmar ^[^^170^^–^^172^^]^	50	2014	$1,408	LM		Gov. Org.
Nepal ^[^^173^^]^	50	2005	$1,071	LM		Gov. Org.
North Korea	NA		NA	Low		
Oman ^[^^145^^,^^174^^]^	10	2012	$15,343	High	GCC	Standards Org.
Pakistan ^[^^175^^]^	50	2010	$1,285	LM		Gov. Org.
Philippines ^[^^176^^]^	10	2016	$3,485	LM		Gov. Org.
Qatar ^[^^145^^,^ ^177^^]^	10	2014	$62,088	High	GCC	Gov. Org.
Saudi Arabia ^[^^145^^,^ ^178^^]^	10	2015	$23,140	High	GCC	Gov. Org.
Singapore ^[^^179^^]^	10	2019	$65,233	High		Law/Decree
South Korea ^[^^180^^,^ ^181^^]^	10	2015	$31,846	High		Gov. Org.
Sri Lanka ^[^^182^^]^	50	2019	$3,853	LM		Law/Decree
Syria ^[^^183^^–^^185^^]^	50	2007	NA	Low		Standards Org.
Taiwan ^[^^186^^]^	10	2017	NA	High		Gov. Org.
Tajikistan ^[^^143^^,^ ^187^^]^	50	1985	$871	LM	CIS	Standards Org.
Thailand ^[^^188^^,^ ^189^^]^	50	2008	$7,807	UM		Gov. Org.
Timor-Leste ^[^^18^^,^ ^190^^]^^i^	10	2017	$577	LM	WHO	Gov. Org.
Turkey ^[^^191^^,^ ^192^^]^	10	2019	$9,127	UM		Law/Decree
Turkmenistan ^[^^193^^–^^195^^]^	NA		NA	UM		
United Arab Emirates ^[^^145^^,^ ^196^^]^^j^	10	2014	$43,103	High	GCC	Gov. Org.
Uzbekistan ^[^^197^^]^	50	2006	$1,725	LM		Law/Decree
Vietnam ^[^^198^^]^	10	2009	$2,715	LM		Gov. Org.
Yemen ^[^^199^^]^	10	1999	$774	Low		Gov. Org.
**Australia and Oceania**						
Australia ^[^^200^^]^	10	2017	$55,060	High		Gov. Org.
Federated States of Micronesia ^[^^135^^,^ ^201^^]^	10	2018	NA	LM	US EPA	Law/Decree
Fiji ^[^^202^^,^ ^203^^]^^k^	10	2011	$6,176	UM		Standards Org.
Kiribati ^[^^18^^,^ ^24^^]^	10	2017	$1,655	LM	WHO	Gov. Org.
Marshall Islands ^[^^205^^]^	50	1994	NA	UM		Law/Decree
Nauru ^[^^18^^,^ ^24^^]^^l^	10	2017	$11,724	High	WHO	Gov. Org.
New Zealand ^[^^207^^]^	10	2018	$42,745	High		Gov. Org.
Palau ^[^^208^^]^	50	1996	$14,902	High		Gov. Org.
Papua New Guinea ^[^^209^^]^	50	2006	$2,829	LM		Law/Decree
Samoa ^[^^210^^–^^212^^]^	10	2016	$4,209	UM		Gov. Org.
Solomon Islands ^[^^18^^,^ ^213^^]^	10	2017	$2,374	LM	WHO	Law/Decree
Tonga ^[^^18^^,^ ^24^^,^ ^214^^]^^m^	10	2017	$4,903	UM	WHO	Gov. Org.
Tuvalu ^[^^18^^,^ ^215^^]^	10	2017	$4,059	UM	WHO	Gov. Org.
Vanuatu ^[^^216^^]^	10	2019	$3,115	LM		Law/Decree
**Europe**						
Albania ^[^^217^^]^	50	2016	$5,353	UM		Law/Decree
Andorra ^[^^218^^]^^n^	10	2007	$40,886	High		Law/Decree
Austria ^[^^219^^]^	10	2001	$50,138	High	EU	Law/Decree
Belarus ^[^^220^^]^	10	2015	$6,663	UM		Law/Decree
Belgium ^[^^221^^]^	10	2003	$46,421	High	EU	Law/Decree
Bosnia and Herzegovina ^[^^222^^]^	10	2010	$6,109	UM		Law/Decree
Bulgaria ^[^^223^^]^	10	2001	$9,828	UM	EU	Law/Decree
Croatia ^[^^224^^]^	10	2019	$14,936	UM	EU	Law/Decree
Cyprus ^[^^225^^]^	10	2001	$20,815	High	EU	Law/Decree
Czech Republic ^[^^226^^]^	10	2014	$23,495	High	EU	Law/Decree
Denmark ^[^^227^^]^	5	2015	$60,170	High	EU	Law/Decree
Estonia ^[^^228^^]^	10	2002	$23,723	High	EU	Gov. Org.
Finland ^[^^229^^]^	10	2014	$48,783	High	EU	Gov. Org.
France ^[^^230^^,^ ^231^^]^	10	2017	$40,494	High	EU	Law/Decree
Germany ^[^^232^^]^	10	2017	$46,445	High	EU	Law/Decree
Greece ^[^^233^^]^	10	2017	$19,583	High	EU	Law/Decree
Hungary ^[^^234^^]^	10	2002	$16,732	High	EU	Law/Decree
Iceland ^[^^235^^]^	10	2001	$66,945	High		Law/Decree
Ireland ^[^^236^^]^	10	2014	$78,661	High	EU	Law/Decree
Italy ^[^^237^^,^ ^238^^]^	10	2016	$33,228	High	EU	Gov. Org.
Kosovo ^[^^239^^]^	10	2012	$4,345	UM	EU	Law/Decree
Latvia ^[^^240^^]^	10	2017	$17,829	High	EU	Law/Decree
Liechtenstein ^[^^241^^]^	10	2018	NA	High		Law/Decree
Lithuania ^[^^242^^]^	10	2017	$19,602	High	EU	Law/Decree
Luxembourg ^[^^243^^]^	10	2017	$114,705	High	EU	Law/Decree
Malta ^[^^244^^]^	10	2009	$29,821	High	EU	Law/Decree
Moldova ^[^^245^^]^	10	2019	$4,504	LM		Law/Decree
Monaco ^[^^246^^]^	10	2017		High		Law/Decree
Montenegro ^[^^247^^]^	10	2012	$8,909	UM		Law/Decree
Netherlands ^[^^248^^]^	10	2011	$52,331	High	EU	Law/Decree
Norway ^[^^249^^]^	10	2016	$75,420	High		Law/Decree
Poland ^[^^250^^]^	10	2017	$15,693	High	EU	Law/Decree
Portugal ^[^^251^^]^	10	2017	$23,252	High	EU	Law/Decree
Republic of North Macedonia ^[^^252^^]^	10	2018	$5,954	UM		Law/Decree
Romania ^[^^253^^]^	10	2019	$12,920	High	EU	Law/Decree
Russia ^[^^254^^]^	50	2001	$11,774	UM		Gov. Org.
San Marino ^[^^255^^]^	10	2012	NA	High		Law/Decree
Serbia ^[^^256^^]^	10	2019	$7,412	UM		Law/Decree
Slovakia ^[^^257^^]^	10	2006	$19,266	High	EU	Law/Decree
Slovenia ^[^^258^^]^	10	2015	$25,946	High	EU	Law/Decree
Spain ^[^^259^^]^	10	2003	$29,367	High	EU	Law/Decree
Sweden ^[^^260^^]^	10	2017	$51,615	High	EU	Law/Decree
Switzerland ^[^^261^^]^	10	2020	$81,994	High		Gov. Org.
Ukraine ^[^^262^^]^	10	2010	$3,465	LM		Gov. Org.
United Kingdom ^[^^263^^]^^o^	10	2016	$42,330	High		Law/Decree

Abbreviations: CARICOM = Caribbean Community; CIS = Commonwealth of Independent States; EAS = East Africa States; EU = European Union; GCC = Gulf Cooperation Council; Gov. Org. = governmental organization (ministry, agency, department); GDP = gross domestic product; LM = Lower Middle; NA = NA; WHO = World Health Organization; UM = Upper Middle; US EPA (United States Environmental Protection Agency)

^a^ Law/Decree includes legislation and presidential or royal decrees; Gov. Org. includes ministries and governmental agencies; Standards Org. includes standards bureaus and standards agencies.

^b^ Standard withdrawn October 14, 2016 [36].

^c^ Caribbean Community and Common Market (CARICOM) standard for packaged purified drinking water is 50 μg/L [106].

^d^ The official government document lists this value as “1,0 ug/l”, which is a magnitude lower than the contemporaneous WHO value [122, 123]. Since the other water contaminants follow the WHO values, this value is likely a typographical error for “10 ug/l”.

^e^ As standard only currently applies to municipalities of > 500K inhabitants; to municipalities of > 50K inhabitants by 2022 and to all by 2025 [128].

^f^ Applies specifically to bottled water [130].

^g^ The official government document lists this value as “0.5 mg/l”, which is a magnitude higher than a previous WHO value [131, 132]. Since the document references the WHO guidelines, and a former WHO guideline for As was 50 μg/L, this value is likely a typographical error for “0.05 mg/l”.

^h^ Permissible limit in the absence of alternative source: 50 μg/L [152].

^i^ Author suggests that East Timor uses WHO guidelines as local standards [190].

^j^ Standards are specifically for the Emirate of Abu Dhabi [196].

^k^ The maximum contaminant level for As in bottled water is 50 μg/L [204].

^l^ A Nauru government document states that there are no standards [206], but the 2018 WHO survey noted that Nauru uses WHO guidelines as standards [18, 24].

^m^ A secondary source states that there are no national standards [214], but the 2018 WHO survey noted that Tonga uses WHO guidelines as standards [18, 24].

^n^ The decree states that in some cases 50 μgl/L is the maximum allowable threshold taking into account the detection limit of the analytical equipment [218].

^o^ Applies specifically to private water supplies [263].

### 3.1 Arsenic regulations

We found evidence of regulations for a maximum allowable concentration of As in drinking water for 176 countries. There were 19 countries for which we could not find any evidence of drinking water regulations. By comparison, in a 2015 survey of global drinking water regulations, the WHO found regulations for As in 102 out of 104 countries [24] while the International Water Resources Association’s 2018 comparison of water quality guidelines included drinking water standards for 10 countries [25].

Of the As regulations that we found, the lowest maximum allowable concentration of As was 1 μg/L, the highest was 500 μg/L, and the mode was 10 μg/L. The lowest maximum allowable concentration for As of 1 μg/L may be a typographical error in the government document establishing this allowable concentration since the other contaminants listed in the document followed the values listed in the WHO’s drinking water guidelines at the time [122, 123]. The highest maximum allowable concentration of 500 μg/L was also likely a typographical error in the official government record [131]. If these 2 likely errors are set aside, the lowest maximum concentration for As was 5 μg/L and the highest maximum concentration was 300 μg/L. When the likely typographical errors are adjusted to their probable intended values, 2 countries have a maximum allowable concentrations less than 10 μg/L, 134 countries have maximum allowable concentrations of 10 μg/L, and 40 countries have maximum allowable concentrations of greater than 10 μg/L. For all statistical analyses that follow, we retained the published regulatory values without adjustments for possible typographical errors.

**Fig 1:**
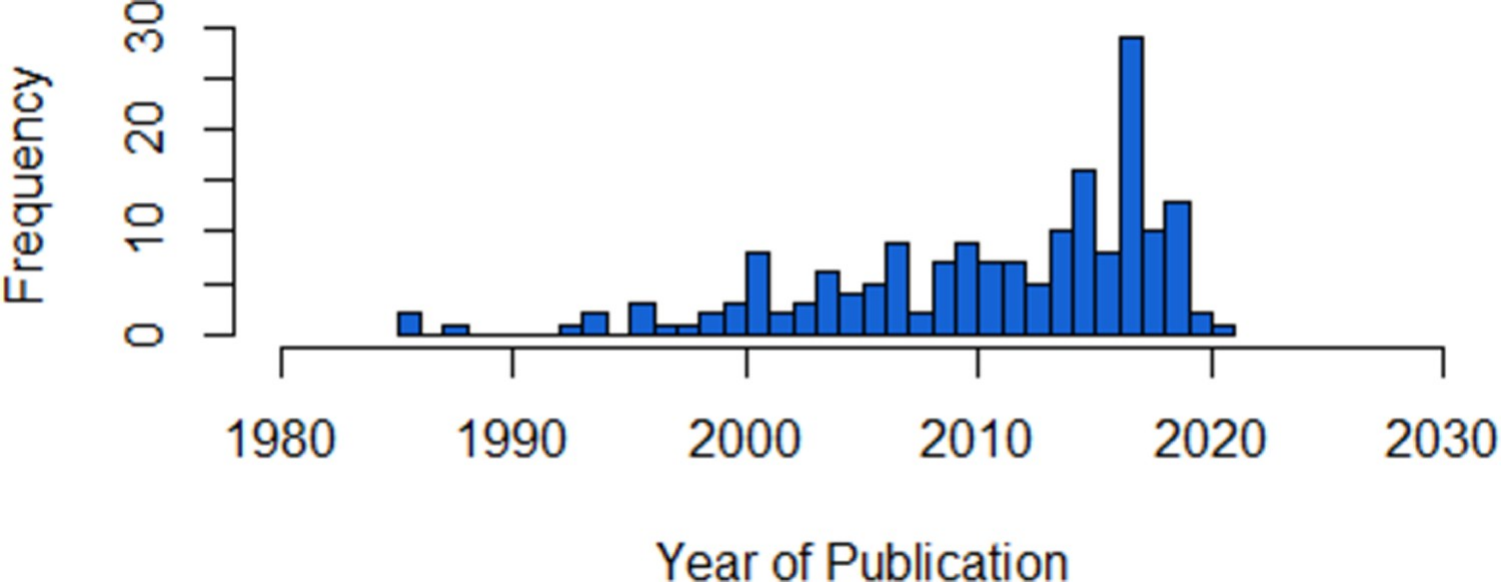
Comparison of publication dates of arsenic drinking water standards.

The oldest regulation for As in drinking water currently in force is dated 1985 [142, 143, 187], while the most recent regulation dates to 2021 [112]. The average date of the regulations is 2011, while the mode is 2017 (Fig 1). Arsenic standards are established by national legislation or decree in 85 countries, ministries or agencies in 58 countries, and national standards boards in 33 countries. Many standards published by national standards boards are copyrighted documents and must be purchased or licensed for a fee in order to be accessed.

### 3.2 Arsenic regulations, population, and per capita income

**Fig 2:**
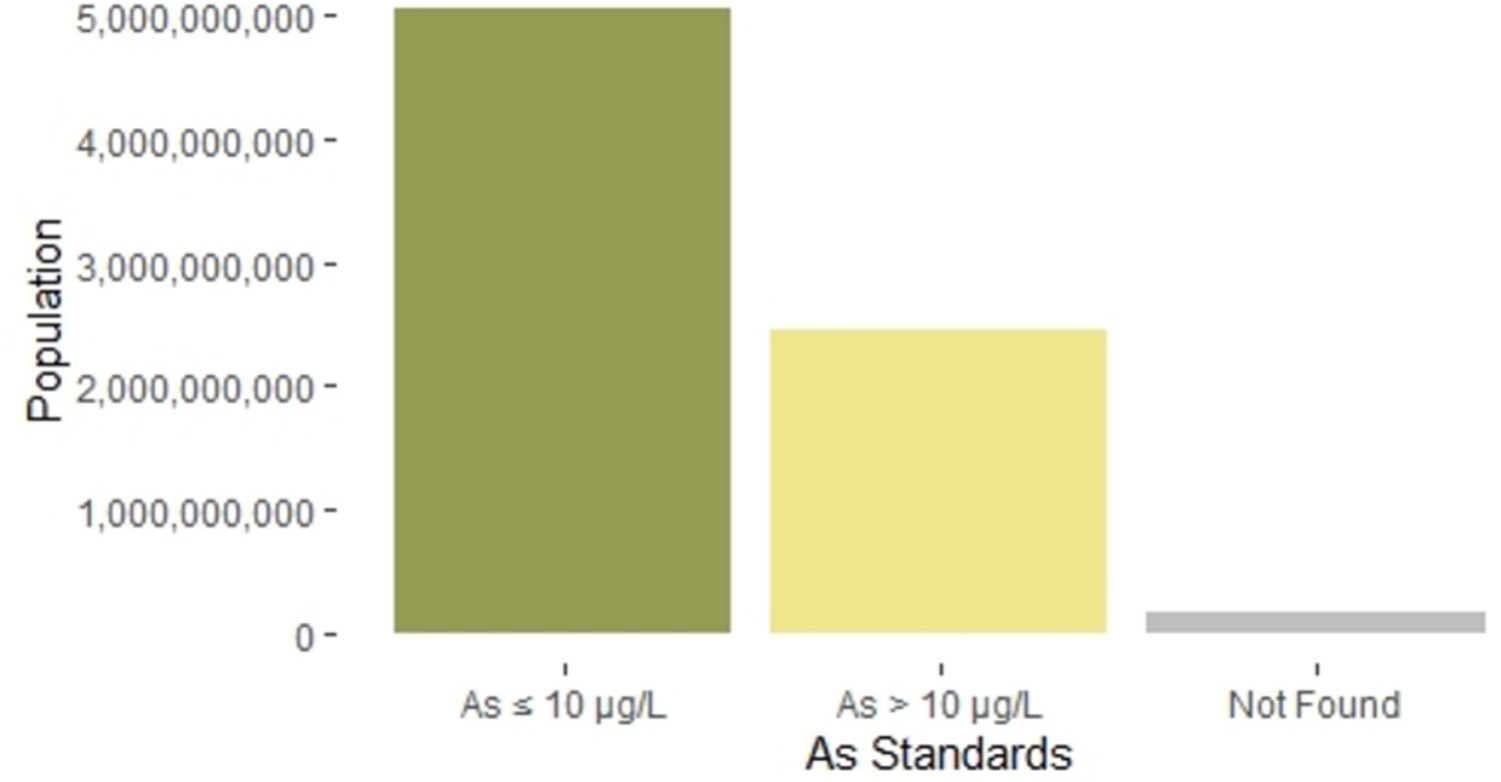
Comparison of populations covered by different levels of arsenic drinking water standards.

Seventy percent (136) of the 195 countries in our survey have regulations for As in drinking water that are equal to or more protective than the WHO’s drinking water guideline of 10 μg/L, while 21% (40) of the 195 countries have regulations that are less protective than the WHO’s 10 μg/L guideline, and we could not find regulations for 10% (19) of the 195 countries. Sixty-six percent of the world’s population lives in countries with As drinking water regulations equal to or more protective than the WHO’s 10 μg/L guideline, 32% live in countries with regulations that are less protective than the WHO’s 10 μg/L guideline, and 2% live in countries where we could not find evidence of a drinking water guideline for As (Fig 2).

Arsenic regulations are also strongly tied to national income as represented by GDP per capita. The sum of all GDPs of the countries with 2019 GDP data and an As regulation equal to or more protective than the WHO guideline of 10 μg/L divided by the sum of the population of these countries was $13,587 per capita. In contrast, the sum of all GDPs of countries with 2019 GDP data and an As regulation less protective than the WHO guideline divided by the sum of the population of these countries was $7,601 per capita. The sum of all GDPs of countries for which we had 2019 GDP data but could not find an As regulation divided by the sum of the population of these countries was $1,227 per capita. The GDPs per capita of countries with As regulations equal to or more protective than the WHO guideline of 10 μg/L were significantly higher (*n* = 129, *M* = $17,678) than those of countries with As regulations less protective than the current 10 μg/L WHO guideline (*n* = 36, *M* = $5,384) (F(2,177) = 7.55, *p* < .001).

**Fig 3:**
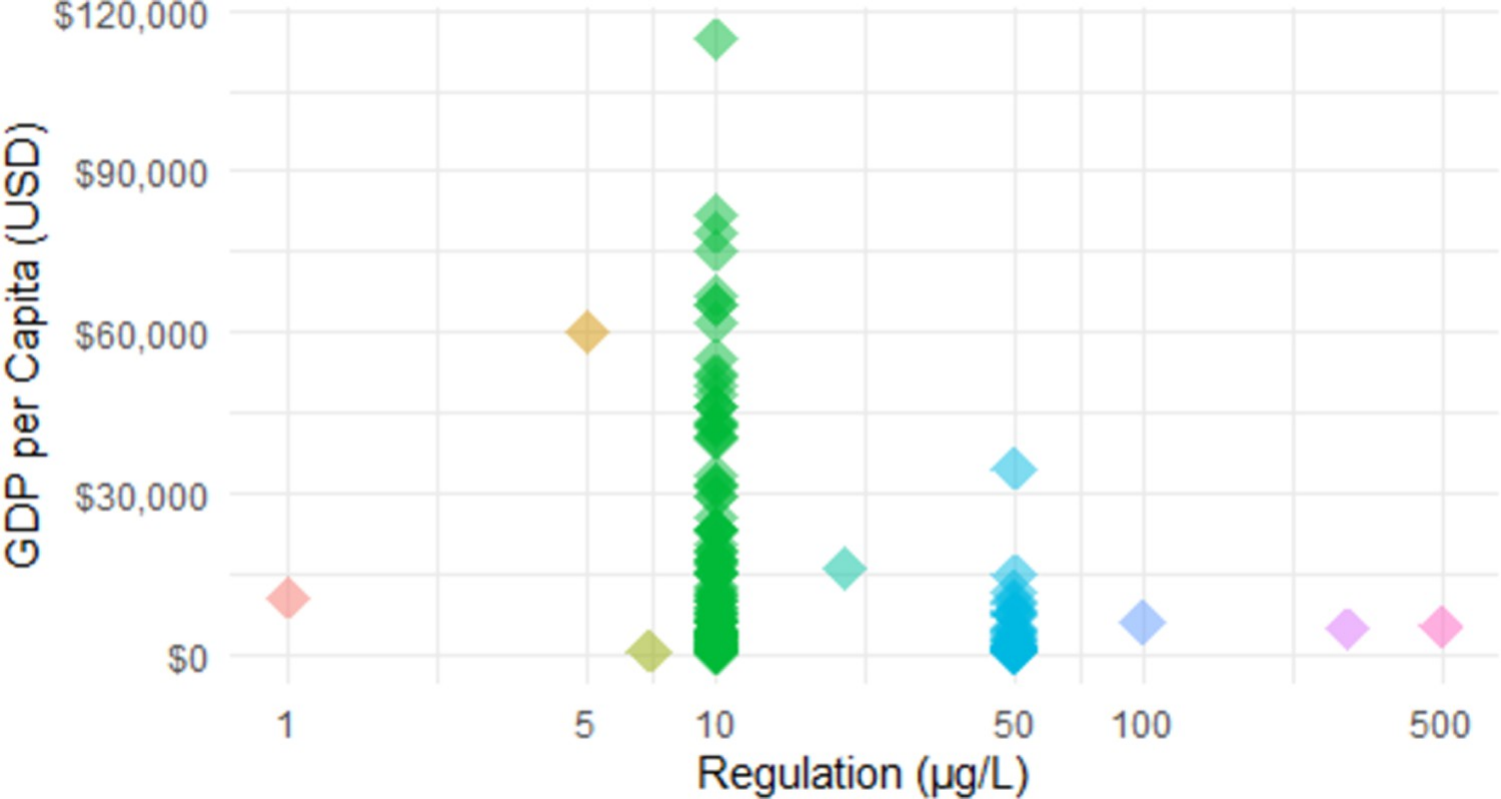
Gross domestic product (GDP) per capita in current (2019) United States dollars (US $) versus national drinking water standard or guideline for arsenic in micrograms per liter (μg/L) for 180 countries [20].

**Fig 4:**
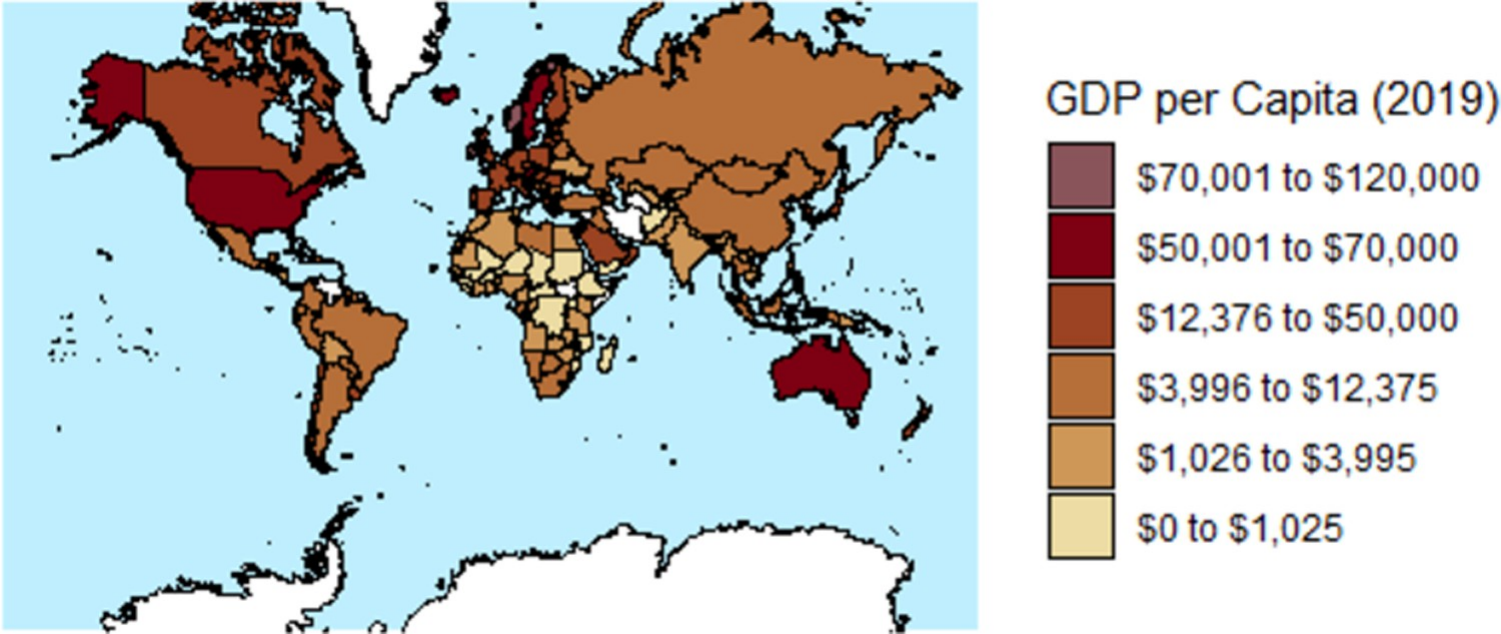
A map of 2019 gross domestic product (GDP) per capita in United States dollars (US $) for 180 countries (map base: [264]).

**Fig 5:**
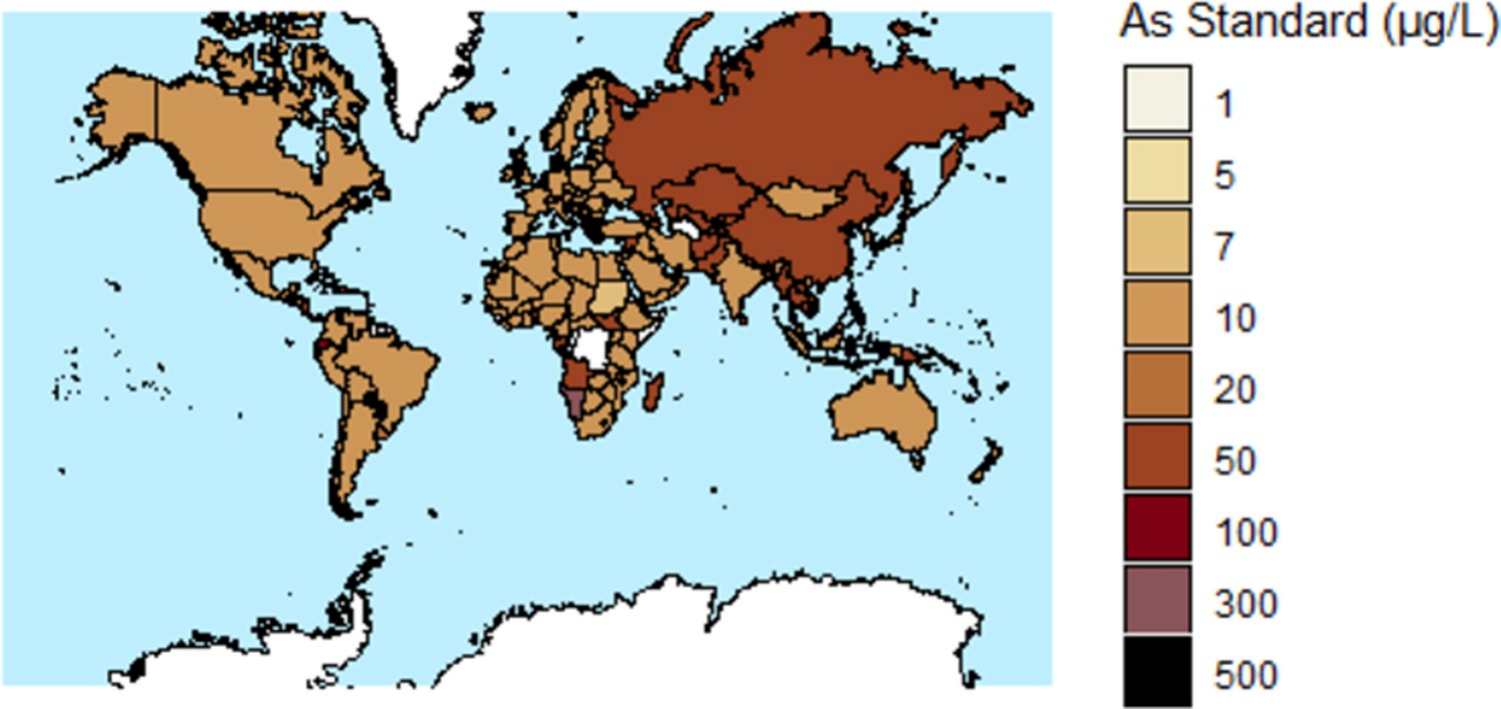
A map of national drinking water standards or guidelines for arsenic in micrograms per liter (μg/L) for 180 countries (map base: [264]).

A graph of GDP per capita versus national drinking water standard or guideline for As is shown in Fig 3. Maps of GDP per capita and national drinking water standards or guidelines for As are shown in Figs 4 and 5, respectively.

**Fig 6:**
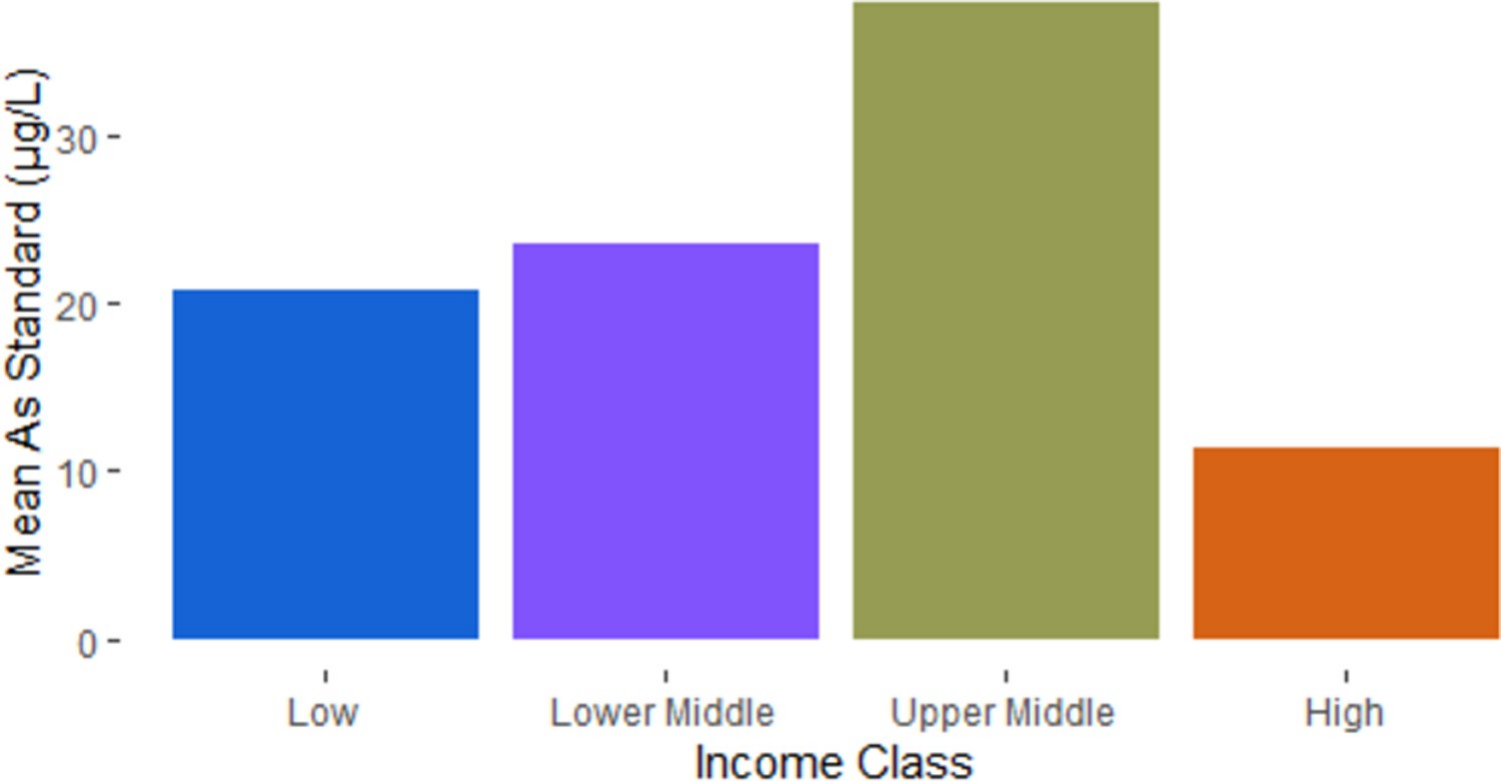
Comparison of mean national arsenic drinking water standards by income group.

The World Bank classifies economies into one of 4 groups: low income, lower middle income, upper middle income, and high income [31]. We found no difference in recency of As regulations by income group (F(3,175) = 0.63, p = 0.60). For the countries that have As regulations, the mean As regulation for low income countries was 21 μg/L, for lower middle income countries the mean was 24 μg/L, for upper middle income countries the mean was 38 μg/L, and for high income countries the mean was 11 μg/L (Fig 6). An analysis of variance (ANOVA) on these means yielded significant variation between income classes (F(3, 172) = 3.15, p = .03). A post hoc Tukey test showed that the mean As standard of the high income class differed significantly from that of the upper middle income class (p = .01) while the remaining differences were not significant (p>0.05).

Sixteen countries tie their maximum allowable As concentrations directly to the WHO drinking water guidelines. A large number of other countries have maximum allowable As concentrations that equal the current or previous WHO drinking water guidelines. This underscores the importance of the WHO drinking water guidelines for protecting the health of world citizens.

### 3.3 The drinking water guideline set by the World Health Organization

The WHO first established a drinking water standard for As, 200 μg/L, in 1958 based on health concerns [123, 265]. As adverse health effects from As exposure received more study, this As drinking water standard was lowered to 50 μg/L in 1963 [16, 123]. In 1971, the World Health Organization (WHO) deemed its 50 μg/L As standard a “tentative limit” [132] and noted, “Some epidemiological studies have suggested that arsenic is carcinogenic but no real proof of the carcinogenicity to man of arsenic in drinking-water has been forthcoming. It would seem wise to keep the level of arsenic in drinking-water as low as possible” [132].

In 1971, 50 μg/L was “as low as possible” for a limit because the available methods for the routine analysis of As in drinking water were not accurate, precise, sensitive, or affordable enough to reliably measure lower concentrations [132]. For example, the recommended method of polarographic estimation was often not accurate due to interferences, and the incomplete reduction of analyte [132, 266]. Similarly, the commonly used spectrophotometric determination of As by the silver diethyldithiocarbamate (AgSCSN(CH_2_CH_3_)_2_) method was not precise due to the sometimes incomplete generation of arsine gas (AsH_3_(g)) from the sample matrix [132, 267]. In contrast, atomic absorption spectroscopy (AAS) was accurate, precise, and sensitive, but not affordable in many low-income countries [132, 267].

In 1984, the WHO began publishing drinking-water “guidelines” instead of “standards” [268]. Guidelines were intended to provide guidance to national regulators and stakeholders, but they are specifically not to be taken as international standards. National regulators are encouraged to take local conditions, resources, and hazards into account when setting national standards [268, 269]. The 1984 WHO drinking-water guideline for As was maintained at 50 μg/L, the former WHO standard [268].

In 1993, the WHO replaced its earlier “tentative limit” of 50 μg/L with a “provisional guideline” of 10 μg/L for As in drinking water [17]. By this time, a skin cancer risk in humans was known, and other cancer risks were suspected. “Inorganic arsenic is a documented human carcinogen and has been classified by IARC [International Agency for Research on Cancer] in Group 1 [‘This category is used when there is *sufficient evidence* of carcinogenicity in humans.’]. A relatively high incidence of skin and possibly other cancers that increase with dose and age has been observed in populations ingesting water containing high concentrations of arsenic” [17]. The WHO calculated a health-based value of 0.17 μg/L, but noted that this value was below the practical quantification limit of 10 μg/L [17]. Therefore, the 10 μg/L drinking water guideline for As was initially “provisional” because of what is now called “analytical achievability” [17, 269]. Thus, the 1993 WHO 10 μg/L drinking water guideline for As was based on practical analytical concerns rather than health data, since health data would have led to a lower guideline [17].

The WHO has maintained this 10 μg/L provisional drinking water guideline for As in all subsequent editions and addendums of *Guidelines for Drinking-water Quality* since 1993 [18, 123, 270–272]. In 2006, the WHO stated that As in drinking water not only causes skin cancer, but also causes bladder and lung cancers [271]. “There is overwhelming evidence from epidemiological studies that consumption of elevated levels of arsenic through drinking-water is causally related to the development of cancer at several sites, particularly skin, bladder and lung” [271]. In 2011, the WHO added kidney cancer to this list [272]. “The International Programme on Chemical Safety (IPCS) concluded that long-term exposure to arsenic in drinking-water is causally related to increased risks of cancer in the skin, lungs, bladder and kidney, as well as other skin changes, such as hyperkeratosis and pigmentation changes” [272]. However, the WHO did not lower their drinking water guideline for As “because [the] calculated guideline value is below the achievable quantification level” [272]. Again, “guideline values are not set at concentrations of substances that cannot reasonably be measured. In such circumstances, provisional guideline values are set at the reasonable analytical limits” [269, 272].

In 2011, the WHO added a second provision to its provisional 10 μg/L drinking water guideline for As [272]. This second provision is based on “treatment performance” [272]. More specifically, “It is technically feasible to achieve arsenic concentrations of 5 μg/l or lower using any of several possible treatment methods. However, this requires careful process optimization and control, and a more reasonable expectation is that 10 μg/l should be achievable by conventional treatment (e.g. coagulation)” [272]. In 2017, the WHO maintained its 10 μg/L provisional drinking water guideline for As, the “analytical achievability” provision for this guideline, and the “treatment performance” provision for this guideline [18, 269].

In summary, the current WHO 10 μg/L provisional drinking water guideline for As is not set according to a health-based risk assessment, since this would require a lower maximum concentration, but rather, it is based on the detection limits for the routine analysis of As in drinking water, which have not been updated since 1993, and treatment technologies for As, which have not been updated since 2011 [18, 272].

### 3.4 Health-based drinking water guidelines

In 2000, the United States Environmental Protection Agency (U.S. EPA) proposed a non-enforceable health-based goal, or Maximum Contaminant Level Goal (MCLG) for As of 0 μg/L [273]. This 0 μg/L MCLG remains in force [135]. In 1999, the U.S. EPA noted that there was a Practical Quantitation Limit (PCL) for As of 3 μg/L [274]. However, instead of using this PQL as the enforceable standard Maximum Contaminant Level (MCL), 5 μg/L was proposed as a national standard based on a cost/benefit analysis [273]. After this proposed MCL of 5 μg/L was set out for public comment, the MCL was raised to 10 μg/L before being adopted as the national standard, over the objections of the U.S. EPA’s own Scientific Advisory Board, who argued for a lower MCL [275, 276].

In contrast, the California Environmental Protection Agency (CalEPA) has set a public health goal (PHG) of 0.004 μg/L for As in drinking water [7, 8]. This PHG is based on human health effects; it is not influenced by detection limits or the performance of drinking water treatment systems. Under California law, a PHG must be based on current scientific evidence and give a negligible risk of adverse health effects over a lifetime of exposure [277]. In addition, a PHG must not consider economic, technical, or other societal factors [277]. As a result, the CalEPA 0.004 μg/L PHG is 2,500 times lower than the WHO provisional drinking water guideline and the U.S. EPA drinking water standard of 10 μg/L.

More specifically, the CalEPA PHG is based on the risk of death from lung and bladder cancers after a lifetime of exposure to As in drinking water [8]. These risks were calculated from epidemiological studies in Taiwan, Chile, and Argentina (Eqs 1, 2, 3, 4, 5 and 6) [8]. Since the risk of death from lung and bladder cancers is greater than that from skin cancer, kidney cancer, and noncancer health effects, the risks from these 3 less significant effects were not factored into the CalEPA PHG (Eqs 1 and 2) [8].


$$\frac{y\;Excess\;Cancer\;Deaths\;}{1,000,000\;People}=\frac{x\;\mu g}{L}\times 0.00024_{75}$$ (Eq 1)

The nonsignificant digits, “75”, are shown as a subscript (Eqs 1, 2, 3, 4, 5 and 6). These nonsignificant digits are included in all steps of a calculation to prevent rounding error. The CalEPA rounded their PHG to 1 significant figure as follows (Eq 2) [8].


$$\frac{0.004\;\mu g}{L}\left(\begin{array}{c} Rounded\;to\\ 1\;Figure \end{array}\right)=\frac{\left(\frac{1\;Excess\;Cancer\;Death\;}{1,000,000\;People}\right)}{\left(0.00024_{75}\right)}$$ (Eq 2)

This 0.004 μg/L PHG is estimated to result in 1 excess cancer death in 1,000,000 people (Eq 2) [8]. That is, if 1,000,000 people drank water with 0.004 μg of As/L over their lifetimes, it is estimated that 1 of these 1,000,000 people would die from cancer because of this exposure to As. The excess death could be prevented if the dose of the carcinogen is lowered or eliminated [8].

In contrast, the 10 μg/L value used as the WHO provisional drinking water guideline and the U.S. EPA MCL is estimated to result in 2,500 excess cancer deaths in 1,000,000 people (Eq 3), or in 1 excess cancer death in 400 people (Eq 4).


$$\frac{2,500\;Excess\;Cancer\;Deaths\;}{1,000,000\;People}\left(\begin{array}{c} Rounded\;to\\ 2\;Figures \end{array}\right)=\frac{10\;\mu g}{L}\times 0.00024_{75}$$ (Eq 3)

$$\frac{1\;Excess\;Cancer\;Death\;}{400\;People}\left(\begin{array}{c} Rounded\;to\\ 2\;Figures \end{array}\right)=\frac{10\;\mu g}{L}\times 0.00024_{75}$$ (Eq 4)

By comparison, the National Research Council estimated that drinking 10 μg/L of As over a lifetime results in 3,700 excess cancer deaths in 1,000,000 males and 3,000 excess cancer deaths in 1,000,000 females (Eq 3) [8].

The 50 μg/L value still used by many countries as national drinking water standards is estimated to result in 12,000 excess cancer deaths in 1,000,000 people (Eq 5), or 1 excess cancer death in 81 people (Eq 6).


$$\frac{12,000\;Excess\;Cancer\;Deaths\;}{1,000,000\;People}\left(\begin{array}{c} Rounded\;to\\ 2\;Figures \end{array}\right)=\frac{50\;\mu g}{L}\times 0.00024_{75}$$ (Eq 5)

$$\frac{1\;Excess\;Cancer\;Death\;}{81\;People}\left(\begin{array}{c} Rounded\;to\\ 2\;Figures \end{array}\right)=\frac{50\;\mu g}{L}\times 0.00024_{75}$$ (Eq 6)

### 3.5 Low-cost methods for improving public health by reducing arsenic exposure

#### 3.5.1 Advances in inexpensive analytical chemistry methods

One reason for using a 50 μg/L drinking water standard for As instead of the more protective WHO 10 μg/L guideline is the high cost of atomic absorption spectrometers or other sophisticated instruments for measuring total As to 10 μg/L or lower. However, recent developments in analytical methods now make it possible to quantify As to 10 μg/L or lower without expensive equipment.

#### 3.5.2 Spectrophotometry

One example of a low-cost method for quantifying As to 10 μg/L or lower without expensive equipment is the arsenomolybdate method, validated in 2005 [267]. By design, the arsenomolybdate method uses the same equipment as the commonly used silver diethyldithiocarbamate (AgSCSN(CH_2_CH_3_)_2_) method for measuring As [267, 278]. In the arsenomolybdate method, As is removed from the sample by reduction to arsine gas (AsH_3_(g)), collected in an absorber by oxidation to arsenic acid (H_3_AsO_4_), colorized by a sequential reaction to arsenomolybdate, and quantified by spectrophotometry at 835 nm (nanometers) [267]. The method detection limit is 7 μg/L (Table 2) [267]. This detection limit is intended to equal the “concentration of a substance that can be measured and reported with 99% confidence that the analyte concentration is greater than 0” [279, 280]. In summary, the arsenomolybdate method is more accurate, precise, and environmentally safe than the AgSCSN(CH_2_CH_3_)_2_ method; and it is more accurate and affordable than the graphite furnace atomic absorption spectroscopy (GFAAS) method [267].

**Table 2:** Common drinking water standards, guidelines, and public health goals for total arsenic (As) in micrograms per liter (μg/L), the detection limits for total As in μg/L by spectrophotometry, and the estimated cancer risks at these concentrations (Eqs 1, 2, 3, 4, 5 and 6). These cancer risks are in bold font and rounded to 2 figures.

As concentration	$\frac{\begin{array}{c} Number\;of\;Excess\\ Cancer\;Deaths \end{array}}{1,000,000\;People}$	$\frac{\begin{array}{c} 1\;Excess\\ Cancer\;Death \end{array}}{Number\;of\;People}$
**50 μg/L** (drinking water standard common in lower income countries)	$\frac{\begin{array}{c} 12,000\;Excess\\ Cancer\;Deaths \end{array}}{1,000,000\;People}$	$\frac{\begin{array}{c} 1\;Excess\\ Cancer\;Death \end{array}}{81\;People}$
**10 μg/L** (WHO provisional drinking water guideline; drinking water standard common in higher income countries)	$\frac{\begin{array}{c} 2,500\;Excess\\ Cancer\;Deaths \end{array}}{1,000,000\;People}$	$\frac{\begin{array}{c} 1\;Excess\\ Cancer\;Death \end{array}}{400\;People}$
**7.5 μg/L** (detection limit by spectrophotometry using suspended nanoparticles [286])	$\frac{\begin{array}{c} 1,900\;Excess\\ Cancer\;Deaths \end{array}}{1,000,000\;People}$	$\frac{\begin{array}{c} 1\;Excess\\ Cancer\;Death \end{array}}{540\;People}$
**7 μg/L (**detection limit by spectrophotometry using arsenomolybdate [267])	$\frac{\begin{array}{c} 1,700\;Excess\\ Cancer\;Deaths \end{array}}{1,000,000\;People}$	$\frac{\begin{array}{c} 1\;Excess\\ Cancer\;Death \end{array}}{580\;People}$
**4 μg/L (**detection limit by spectrophotometry using an arsenoantimonomolybdenum blue-malachite green complex [283])	$\frac{\begin{array}{c} 990\;Excess\\ Cancer\;Deaths \end{array}}{1,000,000\;People}$	$\frac{\begin{array}{c} 1\;Excess\\ Cancer\;Death \end{array}}{1,000\;People}$
**4 μg/L (**detection limit by spectrophotometry using suspended microparticles [284])	$\frac{\begin{array}{c} 990\;Excess\\ Cancer\;Deaths \end{array}}{1,000,000\;People}$	$\frac{\begin{array}{c} 1\;Excess\\ Cancer\;Death \end{array}}{1,000\;People}$
**0.5 μg/L (**detection limit by spectrophotometry using suspended nanoparticles [285])	$\frac{\begin{array}{c} 120\;Excess\\ Cancer\;Deaths \end{array}}{1,000,000\;People}$	$\frac{\begin{array}{c} 1\;Excess\\ Cancer\;Death \end{array}}{8,100\;People}$
**0.3 μg/L (**detection limit by spectrophotometry using a molybdoarsenate-malachite green complex [282])	$\frac{\begin{array}{c} 74\;Excess\\ Cancer\;Deaths \end{array}}{1,000,000\;People}$	$\frac{\begin{array}{c} 1\;Excess\\ Cancer\;Death \end{array}}{13,000\;People}$
**0.004 μg/L** (Public Health Goal Set by the California Environmental Protection Agency [8])	$\frac{\begin{array}{c} 1.0\;Excess\\ Cancer\;Death \end{array}}{1,000,000\;People}$	$\frac{\begin{array}{c} 1\;Excess\\ Cancer\;Death \end{array}}{1,000,000\;People}$

Other advances in inexpensive analytical chemistry methods include the use of cationic dyes, such as malachite green (C_6_H_5_C(C_6_H_4_N(CH_3_)_2_)_2_^+^), that react with oxyanions, such as derivatized As and derivatized phosphorus (P), to form an ionic solid. These solids are either suspended with a surfactant or dissolved with an organic solvent and measured by spectrophotometry [281–283]. More specifically, one method for the determination of total As uses an arsenoantimonomolybdenum blue-malachite green complex [283]. This complex is suspended with Triton™ X-350 and analyzed at 640 nm [283]. The detection limit is 4 μg/L and is defined as the concentration of standard solution that has a 0.01 absorbance (Table 2) [283]. This method is subject to interferences unless the As is removed from the sample by reduction to AsH_3_ gas before color development [283]. Another method for the determination of total As uses a molybdoarsenate-malachite green complex [282]. This complex is concentrated by filtration onto a nitrocellulose membrane filter [282]. This complex and filter are dissolved with 2-methoxyethanol (methyl cellosolve; CH_3_OCH_2_CH_2_OH), and the filtrate is analyzed at 627 nm [282]. The detection limit is 0.3 μg/L; however, the criteria used to calculate this detection limit was not given (Table 2) [282]. Interference from phosphate is corrected by a selective reduction procedure, interference from ferric iron (Fe(III)) is corrected by a cation exchange procedure, and interference from silicate is corrected by an acidic digestion procedure [282]. Alternatively, all of these interferences are corrected if As is removed from the sample by reduction to AsH_3_ gas before color development.

More recently, another cationic dye, ethyl violet (C(C_6_H_4_N(CH_2_CH_3_)_2_)_3_^+^), was reacted with derivatized oxyanions of As to form suspended particles of ionic complexes [284, 285]. More specifically, one method for the determination of total As uses a molybdoarsenate-ethyl violet complex [284]. This complex forms suspended microparticles and an apparently homogenous blue solution [284]. Prior to color development, interference from phosphate is corrected by an anion exchange procedure, and interference from silica is corrected by reaction with sodium fluoride (NaF) [284]. After color development, the excess dye is converted to a colorless carbinol species in strong acid and the molybdoarsenate-ethyl violet complex is analyzed at 612 nm [284]. This decolorization of excess dye significantly reduces the absorbance of reagent blanks and permits a 4 μg/L detection limit (Table 2) [284]. This detection limit is defined as 3σ/*m*, “where σ is the standard deviation of 5 measurements of the reagent blank, and *m* is the slope of the calibration graph” [284]. This method was modified and uses ethyl violet, an isopolymolybdate-iodine tetrachloride complex, and molybdoarsenate to form suspended nanoparticles that are analyzed at 550 nm as a determination of total As [285]. Prior to color development, interference from ferric iron is corrected by reaction with ethylenediaminetetraacetic acid (EDTA), interference from phosphate is corrected by an anion exchange procedure, and interference from silica is corrected by reaction with NaF [285]. The detection limit is 0.5 μg/L (Table 2) [285]. This detection limit is defined as 3σ/*m* [285].

Another method for the determination of total As uses a reduction and selective extraction of arsenite, As(III), into an ionic liquid functionalized with gold (Au) nanoparticles that are analyzed visually or potentially with a spectrophotometer [286]. The reducing agent is ascorbic acid (C_6_H_8_O_6_) [286]. The ionic liquid is prepared by mixing ultrapure water (in this case, resistivity = 18.3 MΩ⋅cm), tetradecyl (trihexyl) phosphonium chloride (Cyphos® IL-101; [C_14_(C_6_)_3_P]Cl), and Triton™ X-114 [286]. This ionic liquid is functionalized by adding chloroauric acid (HAuCl_4_) and potassium tetrahydroborate (KBH_4_) [286]. The functionalized ionic liquid is red in the absence of As(III) and blue in the presence of As(III) [286]. The estimated detection limit by naked eye is 7.5 μg/L (Table 2) [286]. This method is highly selective; 1.0 micromolar (μM) concentrations of K^+^, Na^+^, Ca^2+^, Mg^2+^, Al^3+^, Ni^2+^, Fe^3+^, Cr^3+^, Zn^2+^, Mn^2+^, Pb^2+^, Cd^2+^, Hg^2+^, SO_4_^2−^, PO_4_^3−^, CO_3_^2−^, NO_2_^−^, and SiO_3_^2−^ do not significantly interfere [286]. Higher, but unspecified concentrations, of Cl^−^, NO_3_^−^, and SCN^−^ do not significantly interfere [286].

#### 3.5.3 Summary of advances in inexpensive analytical chemistry methods

In summary, no country needs to use the less protective 50 μg/L standard or guideline due to the expense of analytical chemistry methods. There are many affordable methods for measuring total As to the more protective WHO 10 μg/L provisional drinking water guideline, or to concentrations as low as 0.3 μg/L (Table 2).

#### 3.5.4 Advances in inexpensive drinking water treatment technologies

Another reason for using a 50 μg/L drinking water standard instead of the more protective WHO 10 μg/L guideline is the high expense of drinking water treatment systems. However, advances in inexpensive drinking water treatment technologies have produced technologies that can now remove As to concentrations that are lower than 10 μg/L [287]. Selected examples of these advances follow.

Batch rectors used optimized pH adjustment, oxidation, coagulation, and filtration to economically remove As from drinking water to “about 5 μg/L” during field trials of 10 households and 6 schools in Assam, India [288]. Sodium bicarbonate (NaHCO_3_) was used to adjust pH, potassium permanganate (KMnO_4_) was used to oxidize ambient As(III) and Fe(II) to relatively insoluble As(V) and Fe(III), and iron (III) chloride (FeCl_3_) was used to coagulate the oxidized As and Fe [288]. The treated water was allowed to settle for 1 to 2 hours [288]. The supernatant was filtered through sand-gravel filters [288]. The households each used a 10 L batch rector, 5 schools each used a 25 L batch reactor, and 1 school used a 200 L batch reactor [288]. The concentration of As in the influent ranged from 100 μg/L to 240 μg/L [288]. The concentration of As in the effluent was less than 5 μg/L if the initial concentration of Fe in the influent was less than 1,000 μg/L, and the concentration of As in the effluent was less than 8 μg/L if the initial concentration of Fe in the influent was greater than 2,500 μg/L [288]. The estimated recurring cost per cubic meter (m^3^) of treated water was approximately US $0.16 per/m^3^ [288].

An electrocoagulation batch reactor economically removed As from drinking water to concentrations that were always less than 5 μg/L during a 3.5 month field trial in West Bengal, India [289]. More specifically, electrolytic oxidation of a sacrificial iron (Fe) anode produced Fe(III) precipitates. These Fe(III) precipitates reacted with As in the influent and produced As-Fe(III) precipitates. These As-Fe(III) precipitates aggregated or flocculated together. These flocs were mixed with a small amount of alum and removed by gravitational settling [289]. The concentration of As in the influent was 266 ± 42 μg/L and the average concentration of As in the effluent was 2.1 ± 1.0 μg/L [289]. This batch reactor treated 31,000 L (50 batches) of drinking water during the 3.5 month field trial [289]. The cost of treated water was US $0.83/m^3^ to US $1.04/m^3^ [289].

Above ground adsorbent filters economically removed As from drinking water to concentrations that were consistently less than 10 μg/L during field trials of 20 households and 3 schools in West Bengal, India [290]. These filters were made of activated carbon, charcoal, fine granular sand, activated laterite, and raw laterite [290]. Laterites are highly weathered soils that are dominated by clay sized particles of iron hydrous oxides and aluminum hydrous oxides [291]. The laterite was activated by sequential treatment with hydrochloric acid (HCl) and sodium hydroxide (NaOH) [290]. The concentration of As in the influent ranged from 50 μg/L to 500 μg/L [290]. The concentration of As in the effluent was always less than 10 μg/L [290]. These filters have a relatively large As removal capacity, 32.5 milligrams (mg)/g, and as a result have a relatively long service life, at least 5 years [290]. The cost of the treated water was less than US $0.35/m^3^ [290].

Below ground adsorbent filters economically removed As from drinking water to concentrations that were consistently less than 10 μg/L during field trials of 4 households in Hangjinhouqi County, Inner Mongolia, China [292]. These filters were made by mixing 1 mass unit of a locally abundant limestone with 2 mass units of a locally abundant Fe-mineral (hematite and goethite) [292]. This mixture was placed below ground, around the well screen of a conventional tube well [292]. The limestone likely increased the pH of the groundwater [293]. This increase in pH likely enhanced the oxidation of soluble As(III) with dissolved oxygen (O_2_(g)) to make insoluble As(V) [294]. This As(V) was removed from the groundwater by precipitation with the dissolved calcium (Ca^2+^(aq)) from the limestone and by adsorption to the surface of the Fe-mineral [292]. The concentration of As in the unfiltered groundwater ranged from 318 μg/L to 635 μg/L [292]. The concentration of As in the filtered groundwater was always less than 10 μg/L [292]. “The filtration system was continuously operated for a total volume of 365,000 L, which is sufficient for drinking water supplying a rural household of 5 persons for 5 years at a rate of 40 L per person per day” [292]. The cost of the treated water was less than US $0.10/m^3^ [292].

A groundwater extraction, aeration, and reinjection system economically removed As from drinking water to concentrations that were consistently less than 10 μg/L during a village scale field trial in West Bengal, India [295]. More specifically, groundwater was extracted from the aquifer with a submersible electric pump [295]. This groundwater was aerated by spraying it into an above ground plastic tank with “ordinary plastic shower heads” and letting it sit in the tank for at least 30 minutes [295]. The final concentration of O_2_(aq) in this aerated water ranged from 4 mg/L to 6 mg/L [295]. Approximately, 15% to 20% of this aerated water as reinjected into the aquifer by gravity, and the remaining 80% to 85% of this aerated water was used for drinking [295]. The concentration of As in the influent was not clearly specified [295]. The concentration of As in the effluent was always less than 10 μg/L [295]. The cost of the treated water was US $0.50/m^3^ [295].

In summary, no country needs to use the less protective 50 μg/L standard or guideline due to the expense of treatment technologies. There are many affordable methods for treating water to reduce As concentrations to lower than 10 μg/L (Table 3).

**Table 3:** Common drinking water standards, guidelines, and public health goals for total arsenic (As) in micrograms per liter (μg/L), the effluent concentrations of total As in μg/L from water treatment systems used in the low-income world, and the estimated cancer risks at these concentrations (Eqs 1, 2, 3, 4, 5 and 6). These cancer risks are in bold font and rounded to 2 figures.

As concentration	$\frac{\begin{array}{c} Number\;of\;Excess\\ Cancer\;Deaths \end{array}}{1,000,000\;People}$	$\frac{\begin{array}{c} 1\;Excess\\ Cancer\;Death \end{array}}{Number\;of\;People}$
**50 μg/L** (drinking water standard common in lower income countries)	$\frac{\begin{array}{c} 12,000\;Excess\\ Cancer\;Deaths \end{array}}{1,000,000\;People}$	$\frac{\begin{array}{c} 1\;Excess\\ Cancer\;Death \end{array}}{81\;People}$
**10 μg/L** (WHO provisional drinking water guideline; drinking water standard common in higher income countries)	$\frac{\begin{array}{c} 2,500\;Excess\\ Cancer\;Deaths \end{array}}{1,000,000\;People}$	$\frac{\begin{array}{c} 1\;Excess\\ Cancer\;Death \end{array}}{400\;People}$
**<10 μg/L (**treatment performance using above ground adsorbent filters [290]; below ground adsorbent filters [292]; groundwater extraction, aeration, and reinjection system [295])	$\frac{\begin{array}{c} <2,500\;Excess\\ Cancer\;Deaths \end{array}}{1,000,000\;People}$	$\frac{\begin{array}{c} <1\;Excess\\ Cancer\;Death \end{array}}{400\;People}$
**5 μg/L (**treatment performance using an optimized pH adjustment, oxidation, coagulation, and filtration in a batch reactor [288])	$\frac{\begin{array}{c} 1,200\;Excess\\ Cancer\;Deaths \end{array}}{1,000,000\;People}$	$\frac{\begin{array}{c} 1\;Excess\\ Cancer\;Death \end{array}}{810\;People}$
**<5 μg/L** (treatment performance using an electrocoagulation batch reactor [289])	$\frac{\begin{array}{c} <1,200\;Excess\\ Cancer\;Deaths \end{array}}{1,000,000\;People}$	$\frac{\begin{array}{c} <1\;Excess\\ Cancer\;Death \end{array}}{810\;People}$
**0.004 μg/L** (Public Health Goal Set by the California Environmental Protection Agency [8])	$\frac{\begin{array}{c} 1.0\;Excess\\ Cancer\;Death \end{array}}{1,000,000\;People}$	$\frac{\begin{array}{c} 1\;Excess\\ Cancer\;Death \end{array}}{1,000,000\;People}$

### 3.6 Methods for improving public health by reducing arsenic exposure that require higher expenditures

#### 3.6.1 Other advances in analytical chemistry methods

Some countries use a 10 μg/L drinking water standard or guideline because they assume that this continues to be the limit of quantification for routine analytical chemistry laboratories. However, recent advances in analytical chemistry have developed methods that can be used in routine laboratories with detection limits that are at 0.1 μg/L or less [18, 296]. With these advances 10 μg/L As should no longer be considered the practical limit of quantification, so revised As standards or guidelines could be more protective of public health.

#### 3.6.2 Inductively coupled plasma-mass spectrometry

Advances in inductively coupled plasma-mass spectrometry (ICP-MS) give detection limits for total As that are at 0.1 μg/L or less [18, 296]. Moreover, ICP-MS is commonly used in routine drinking water testing laboratories because it can simultaneously measure the concentrations of almost all of the elements on the periodic table. This reduces cost by allowing each sample to be analyzed for multiple elements in just a few seconds.

An ICP-MS ionizes the As or other analytes in an inductively coupled plasma (ICP) and then separates and quantifies these ions in a mass spectrometer (MS) [296]. The drinking water sample is turned into an aerosol and delivered to an argon (Ar) plasma [296]. This plasma is a flame like object at the top of an ICP torch, and has a sufficient concentration of Ar cations (Ar^+^(g)) and free electrons (e^−^(g)) to make the gas electrically conductive (Eq 7) [296].


$${Ar}_{\left(g\right)}⇆{Ar}_{\left(g\right)}^{+}+e_{\left(g\right)}^{-}$$ (Eq 7)

This torch is surrounded by an induction coil [296]. This coil transmits power from a radio frequency (RF) generator to the plasma [296]. This energy maintains the ionization of the Ar gas, and the temperature of the plasma from about 5,500 kelvin (K) to 8,000 K [296]. These temperatures are approximately 2 or 3 times hotter than all flame spectroscopic methods [296]. As a result, these higher temperatures help make ICP-MS more sensitive than flame atomic absorption spectrometry (FAAS), flame atomic emission spectrometry (FAES), and flame atomic fluorescence spectrometry (FAFS) [296]. The reaction for the ionization of As in a plasma follows (Eq 8).


$${As}_{\left(g\right)}⇆{As}_{\left(g\right)}^{+}+e_{\left(g\right)}^{-}$$ (Eq 8)

These Ar^+^(g; Eq 7) ions, As^+^(g; Eq 8) ions, and any other ions exit the ICP and enter the MS [296]. An MS is a mass filter and a mass detector [296, 297]. The most common type of MS used in atomic mass spectroscopy is the quadrupole mass analyzer [296]. This analyzer uses direct current (DC) and radiofrequency (RF) fields to filter ions [296, 297]. These ions are directed in between 4 parallel rods, the quadrupole [296, 296]. These rods are connected to a source of variable DC voltages; 2 rods are positively charged and 2 rods are negatively charged [296]. In addition, variable RF alternating current (AC) voltages are applied to each pair of rods [296]. The DC and AC voltages on the rods are simultaneously changed; however, the ratio of these voltages is held constant [296]. This makes the ions oscillate between the rods [296]. At any instant, only the ions with the desired mass and charge exit the quadrupole and are detected at an ion transducer; all other ions hit the rods, are converted to 0 charge, and are not detected [296]. In this way, an entire spectrum of atomic masses can be scanned in less than 0.1 seconds [296]. Ultimately, the MS detector measures the ratio of mass to charge (*m/z*) for each cation [296–297]. Since ions with multiple charges are rarely produced, the charge (*z*) is normally assumed to equal 1 and *m/z* is normally the mass of the cation [296–297].

The only naturally occurring isotope for As is ^75^As; that is, all of the As in nature has 75 protons and neutrons [298]. Therefore, all of the As in nature has *m/z* = 75. Unfortunately, if a drinking water sample has chlorine (Cl), calcium (Ca), or sulfur (S), these common atoms can react with the plasma to form ^40^Ar^35^Cl^+^(g), ^38^Ar^37^Cl^+^(g), ^37^Cl_2_^1^H^+^(g), ^40^Ca^35^Cl^+^(g), or ^40^Ar^34^S^1^H^+^(g) [299, 300]. These are polyatomic interferences; they all have *m/z* = 75 and cannot be distinguished from ^75^As^+^(g) [299, 300]. No other isotope of As can be used to eliminate this systematic error [298]. Mathematical corrections can be used to estimate the contribution of these polyatomic interferences to the signal at *m/z* = 75; however, the combined uncertainties in these corrections tend to inflate the detection limit [301].

Fortunately, advances in collision/reaction cell (C/RC) technology are being used to eliminate these polyatomic interferences and give lower detection limits [301, 302]. If a polyatomic ion exits an ICP, enters a C/RC, and collides with an inert gas, such as helium (He(g)), the interference can be eliminated by breaking the polyatomic ion apart (collision-induced dissociation, CID) or by slowing the polyatomic ion down (kinetic energy discrimination, KED) [300]. Or if a polyatomic ion exits an ICP, enters a C/RC, and collides with a reactive gas, such as hydrogen (H_2_(g)), the interference can be eliminated by changing the mass of the polyatomic ion [300]. For example, an Agilent Technologies 7500c ICP-MS using a C/RC with 0.5 mL/minute of He(g) and 3.8 mL/minute of H_2_(g) eliminated interferences from 1 g/L of sodium chloride (NaCl) and gave a 0.025 μg/L detection limit for total As (Table 4) [302]. In this case, this detection limit is the lowest concentration that can be quantified at the 99.86% confidence level [302, 303]. This 0.025 μg/L detection limit is 400 times less than a 10 μg/L drinking water standard (Table 4).

**Table 4:** Common drinking water standards, guidelines, and public health goals for total arsenic (As) in micrograms per liter (μg/L), the detection limits for total As in μg/L by inductively coupled plasma-mass spectrometry (ICP-MS), inductively coupled plasma-tandem mass spectrometry (ICP-MS/MS), and hydride generation-gas chromatography-photoionization detection (HG-GC-PID), and the estimated cancer risks at these concentrations (Eqs 1, 2, 3, 4, 5 and 6). These cancer risks are in bold font and rounded to 2 figures.

As concentration	$\frac{\begin{array}{c} Number\;of\;Excess\\ Cancer\;Deaths \end{array}}{1,000,000\;People}$	$\frac{\begin{array}{c} 1\;Excess\\ Cancer\;Death \end{array}}{Number\;of\;People}$
**50 μg/L** (drinking water standard common in lower income countries)	$\frac{\begin{array}{c} 12,000\;Excess\\ Cancer\;Deaths \end{array}}{1,000,000\;People}$	$\frac{\begin{array}{c} 1\;Excess\\ Cancer\;Death \end{array}}{81\;People}$
**10 μg/L** (WHO provisional drinking water guideline; drinking water standard common in higher income countries)	$\frac{\begin{array}{c} 2,500\;Excess\\ Cancer\;Deaths \end{array}}{1,000,000\;People}$	$\frac{\begin{array}{c} 1\;Excess\\ Cancer\;Death \end{array}}{400\;People}$
**0.029 μg/L (**detection limit by ICP-MS using a Knotted Reactor [304])	$\frac{\begin{array}{c} 7.2\;Excess\\ Cancer\;Deaths \end{array}}{1,000,000\;People}$	$\frac{\begin{array}{c} 1\;Excess\\ Cancer\;Death \end{array}}{140,000\;People}$
**0.025 μg/L (**detection limit by ICP-MS using a Collision/Reaction Cell [302])	$\frac{\begin{array}{c} 6.2\;Excess\\ Cancer\;Deaths \end{array}}{1,000,000\;People}$	$\frac{\begin{array}{c} 1\;Excess\\ Cancer\;Death \end{array}}{160,000\;People}$
**0.004 μg/L (**public health goal set by the California Environmental Protection Agency [8])	$\frac{\begin{array}{c} 1.0\;Excess\\ Cancer\;Death \end{array}}{1,000,000\;People}$	$\frac{\begin{array}{c} 1\;Excess\\ Cancer\;Death \end{array}}{1,000,000\;People}$
**0.0016 μg/L (**detection limit by ICP-MS/MS using a collision/reaction cell with O_2_(g) [305])	$\frac{\begin{array}{c} 0.40\;Excess\\ Cancer\;Deaths \end{array}}{1,000,000\;People}$	$\frac{\begin{array}{c} 1\;Excess\\ Cancer\;Death \end{array}}{2,500,000\;People}$
**0.00082 μg/L (**detection limit by HG-GC-PID [307])	$\frac{\begin{array}{c} 0.20\;Excess\\ Cancer\;Deaths \end{array}}{1,000,000\;People}$	$\frac{\begin{array}{c} 1\;Excess\\ Cancer\;Death \end{array}}{4,900,000\;People}$
**0.0002 μg/L (**detection limit by ICP-MS/MS using a collision/reaction cell with 10% CH_3_F(g) and 90% He(g) [306])	$\frac{\begin{array}{c} 0.050\;Excess\\ Cancer\;Deaths \end{array}}{1,000,000\;People}$	$\frac{\begin{array}{c} 1\;Excess\\ Cancer\;Death \end{array}}{20,000,000\;People}$

Another advance uses flow injection analysis (FIA) and a knotted reactor to remove interference precursors from the sample matrix and concentrate total inorganic As before analysis by ICP-MS [304]. Arsenate (As(V)) was reduced to arsenite (As(III)) in a solution of 1% (mass/volume) L-cysteine (HSCH_2_CHNH_2_COOH) and 0.03 molar (M) nitric acid (HNO_3_) [304]. This As(III) was complexed with a solution of 0.1% (mass/volume) ammonium pyrrolidine dithiocarbamate ((CH_2_)_4_NCS_2_NH_4_) [304]. This complex was absorbed on the inner wall of a knotted reactor, in this case a 150-centimeter (cm) long by 0.5-millimeter (mm) inside diameter (ID) piece of polytetrafluoroethylene (PTFE) tubing [304]. The interference precursors from the sample matrix were washed away and the total inorganic As was concentrated when this complex was absorbed in the reactor [304]. This complex was desorbed from the reactor with 1 molar (M) HNO_3_ and eluted into a Perkin-Elmer-Sciex ELAN 5000 ICP-MS [304]. This gave a 0.029 μg/L detection limit for total inorganic As (Table 4) [304]. This detection limit is defined as 3 times the sample standard deviation (*s*), presumably from the measurement of reagent blanks [304].

#### 3.6.3 Inductively coupled plasma-tandem mass spectrometry

Advances in inductively coupled plasma-tandem mass spectrometry (ICP-MS/MS) give detection limits for total As that are 0.01 μg/L or less [301]. The first MS is typically used as a mass filter and lets through only ions at *m/z* = 75 (^75^As^+^(g), ^40^Ar^35^Cl^+^(g), ^38^Ar^37^Cl^+^(g), ^37^Cl_2_^1^H^+^(g), ^40^Ca^35^Cl^+^(g), and ^40^Ar^34^S^1^H^+^(g)) [299–301]. If present, these ions enter a C/RC and the ^75^As^+^(g) selectively reacts with either oxygen (O_2_(g)) or fluoromethane (CH_3_F(g)) to produce ^75^As^16^O^+^(g) at *m/z* = 91 or ^75^As^12^C^1^H_2_^+^(g) at *m/z* = 89, respectively [301, 305, 306]. The second MS is used to filter the interferences at *m/z* = 75, and quantify the ^75^As^16^O^+^(g) at *m/z* = 91 or ^75^As^12^C^1^H_2_^+^(g) at *m/z* = 89 [301, 305, 306]. This use of 2 mass spectrometers in tandem increases sensitivity and lowers the detection limit [301].

For example, if O_2_(g) is used in the C/RC, a 0.0016 μg/L detection limit for total As was observed (Table 4) [306]. This detection limit is defined as the lowest concentration that can be quantified at the 99.7% confidence level [303, 305]. If 10% CH_3_F(g) and 90% He(g) are used in the C/RC, a 0.0002 μg/L detection limit for total As was observed (Table 4) [306]. This detection limit is defined as 3*s*/*m*, where *s* is the sample standard deviation from 10 measurements of a reagent blank, and *m* is the average slope from 10 calibration graphs [306].

ICP-MS/MS is a very new technology; the first commercial instruments were sold in 2012 [301]. As a result, ICP-MS/MS is mostly used for research and is not commonly used in drinking water testing laboratories. However, ICP-MS/MS will likely become more commonly used for drinking water testing in the future.

#### 3.6.4 Hydride generation-gas chromatography-photoionization detection

Advances in hydride generation-gas chromatography-photoionization detection (HG-GC-PID) give a detection limit for total inorganic As at 0.00082 μg/L [307]. This detection limit is defined as 3*s*, where *s* is the sample standard deviation from 5 measurements of a reagent blank [307]. The hydride generation step uses 50 mL of sample, 2.0 mL of concentrated hydrochloric acid (HCl), 3.0 mL of 1.0 M potassium iodide (KI), and 4.0 mL of 4.0% (weight/volume) sodium borohydride (NaBH_4_) to reduce arsenate (As(V)) and arsenite (As(III)) to arsine gas (AsH_3_(g)) [307]. Helium (He(g)) is used as a carrier gas to move the AsH_3_(g) from the reaction vessel, to a trap at −50°C, a trap at −196°C, a gas chromatograph (GC), and a photoionization detector (PID) [307]. If present, water vapor (H_2_O(g)) is an interference and is removed from the AsH_3_(g) in a trap that is cooled to −50°C with dry ice (CO_2_(s)) and 2-propanol (CH_3_CHOHCH_3_) [307]. After this step, the AsH_3_(g) is concentrated in a trap that is cooled to −196°C with liquid nitrogen (N_2_(l)) [307]. If present, stibine gas (SbH_3_(g)) is an interference and is separated from the AsH_3_(g) in a GC with a Carbopack™ B HT column [307]. Finally, AsH_3_(g) is quantified using a 10.2 electron volt (eV) PID [307].

#### 3.6.5 Summary of other advances in analytical chemistry methods

In summary, no country needs to use the less protective 10 μg/L standard or guideline due to the expense of analytical chemistry methods. There are many methods for measuring total As to lower and the more protective concentrations (Table 4).

#### 3.6.6 Other advances in drinking water treatment technologies

Another reason for continuing to use a 10 μg/L drinking water standard is “treatment performance” [18, 272]. However, recent advances in treatment technologies allow removal of As to concentrations that are significantly lower than 10 μg/L and would allow lower standards that would be more protective of public health. Selected examples of these advances follow.

By law, the Netherlands has a 10 μg/L drinking water standard [248]; however, the Netherlands voluntarily uses a less than 1 μg/L drinking water guideline to better protect public health. In 2015, “the Association of Dutch Drinking water Companies (Vewin) voluntarily agreed on a guideline of <1 μg/L for As in drinking water” [308]. “This policy is based on a two-step assessment of As in drinking water, including i) an assessment of excess lung cancer risk for Dutch population and ii) a cost-comparison between the health care provision for lung cancer and As removal from water to avoid lung cancer” [308]. The average concentrations of As in raw water from 241 public supply well fields in the Netherlands ranges from <0.5 μg/L to 69 μg/L; the treatment of this water to <1 μg/L saves the Netherlands from 7.2 million Euros (M€)/year to 14 M€/year [310]. This 7.2 M€/year to 14 M€/year includes the savings in health care costs from not having to treat excess lung cancer cases and the engineering costs from treating drinking water [308].

In the Netherlands, As is typically removed from raw well water by an optimized aeration and rapid sand filtration process [308]. This aeration oxidizes the soluble Fe(II) and As(III) that is naturally found in raw well water to insoluble Fe(III) and As(V) [308]. A soluble Fe(III) coagulant, such as FeCl_3_, is sometimes added to the raw well water [308]. This produces As-Fe(III) precipitates that are removed in a rapid sand filter [308]. This sand filter sometimes uses a coarse granular top layer and finer bottom layer [308]. This optimized process routinely removes As to <1 μg/L [308].

An optimized reverse osmosis process can also economically remove As from drinking water to concentrations that are significantly lower than 10 μg/L [309]. This process uses a 2-stage membrane cascade and can supply drinking water at 0.5 μg of As/L to a population of 20,000 people for US $1,041/day or US $0.52/m^3^ [309]. A 2-stage membrane cascade has 2 reverse osmosis units connected in series. The feed water enters the first stage, the retentate or reject water from the first stage is discarded, and the permeate from the first stage enters the second stage. The retentate from the second stage is added to the feed water of the first stage. The permeate from the second stage is disinfected and distributed as drinking water. This process uses polyamide membranes [309]. The most significant cost is energy consumption; it is 35% of the total cost [309].

In summary, no country needs to use the less protective 10 μg/L standard or guideline due to limitations of treatment technologies. There are many treatment methods for reducing total As concentrations to lower more protective concentrations (Table 5).

**Table 5:** Common drinking water standards, guidelines, and public health goals for total arsenic (As) in micrograms per liter (μg/L), the effluent concentrations of total As in μg/L from water treatment systems used in the high-income world, and the estimated cancer risks at these concentrations (Eqs 1, 2, 3, 4, 5 and 6). These cancer risks are in bold font and rounded to 2 figures.

Drinking Water Standard, Drinking Water Guideline, Public Health Goal, or Effluent Concentration	$\frac{\begin{array}{c} Number\;of\;Excess\\ Cancer\;Deaths \end{array}}{1,000,000\;People}$	$\frac{\begin{array}{c} 1\;Excess\\ Cancer\;Death \end{array}}{Number\;of\;People}$
**50 μg/L** (drinking water standard common in lower income countries)	$\frac{\begin{array}{c} 12,000\;Excess\\ Cancer\;Deaths \end{array}}{1,000,000\;People}$	$\frac{\begin{array}{c} 1\;Excess\\ Cancer\;Death \end{array}}{81\;People}$
**10 μg/L** (WHO provisional drinking water guideline; drinking water standard common in higher income countries)	$\frac{\begin{array}{c} 2,500\;Excess\\ Cancer\;Deaths \end{array}}{1,000,000\;People}$	$\frac{\begin{array}{c} 1\;Excess\\ Cancer\;Death \end{array}}{400\;People}$
**<1 μg/L (**treatment performance using an optimized aeration and rapid sand filtration process [308])	$\frac{\begin{array}{c} <250\;Excess\\ Cancer\;Deaths \end{array}}{1,000,000\;People}$	$\frac{\begin{array}{c} <1\;Excess\\ Cancer\;Death \end{array}}{4,000\;People}$
**0.5 μg/L (**treatment performance using an optimized reverse osmosis 2-stage membrane cascade [309])	$\frac{\begin{array}{c} 120\;Excess\\ Cancer\;Deaths \end{array}}{1,000,000\;People}$	$\frac{\begin{array}{c} 1\;Excess\\ Cancer\;Death \end{array}}{8,100\;People}$
**0.004 μg/L (**public health goal set by the California Environmental Protection Agency [8])	$\frac{\begin{array}{c} 1.0\;Excess\\ Cancer\;Death \end{array}}{1,000,000\;People}$	$\frac{\begin{array}{c} 1\;Excess\\ Cancer\;Death \end{array}}{1,000,000\;People}$

#### 3.7 Implementation of regulations

Unfortunately, having legal standards for drinking water quality does not guarantee that these standards will be implemented, or that efforts will be made by suppliers to meet those standards, especially in regions where government and resources are limited. Implementation of standards can also be complicated when multiple jurisdictions are involved [310]. Transparency is vital for the effectiveness of guidelines and standards to protect public health [310]. In countries where standards bureaus control drinking water standards and release them only for a fee, transparency is severely curtailed. Compliance requires additional efforts and resources.

In contrast, in regions where government is effective and resources are sufficient, such as in Denmark and the Netherlands, reducing the national drinking water standard for As to below 5 μg/L in was found to be both technically feasible and affordable [248, 311]. Even in regions in which there are limited resources for implementation and compliance, establishing and updating drinking water standards and guidelines can be of use to stakeholders who need to determine whether a water source is safe, and to put pressure on water suppliers to improve the quality of the water [311].

## 4. Conclusions

As demonstrated in this study, national and international guidelines and standards for As in drinking water need to be updated in accordance with current research and technologies. Updating regulations has the potential to improve public health by reducing As exposures worldwide. Technologies are now available that could enable both resource-limited and high income countries to adopt more protective drinking water regulations for As.

The WHO 10 μg/L drinking water guideline for As is provisional because it does not sufficiently protect public health. The value of 10 μg/L was specified on the basis of “analytical achievability” or “treatment performance” [17, 18, 132, 272]. However, since the value for this guideline of 10 μg/L for was selected by the WHO in 1993, numerous new technologies and methods have been developed so that 10 μg/L no longer represents a practical limit for either analytical achievability or As treatment performance. This global drinking water guideline should be updated to better protect public health.

Many countries, especially lower income countries continue to use the long-outdated (1963) former WHO standard of 50 μg/L as a maximum allowable concentration for As [16]. Because many of these countries also have high populations, nearly one third of the world’s population lives in jurisdictions with a 50 μg/L standard for As. This concentration of 50 μg/L is associated with high levels of morbidity and mortality and can no longer be justified by the high cost of As quantification or treatment since new low-cost analytical and treatment methods are now available. Lowering the maximum allowable concentration from 50 μg/L to 10 μg/L or lower is urgently needed to avoid countless preventable cancer deaths and to better protect public health.

A variety of solutions are required to update and set more protective national drinking water standards and guidelines for As. In fact, each country might set a hierarchy of drinking water standards and guidelines for As. For example, low-income countries with limited resources might need to rely on relatively inexpensive spectrophotometers for testing and relatively simple systems for treating drinking water. If such a country or parts of a country had As-free water (Figs 2 and 3), then a 50 μg/L standard based on analytical achievability could be lowered to a more protective 0.3 μg/L standard through simple testing and water sharing (Table 2). This would lower the lifetime cancer risk from 12,000 excess cancer deaths in 1,000,000 people (1 excess cancer death in 81 people) to 74 excess cancer deaths in 1,000,000 people (1 excess cancer death in 13,000 people; see Table 2). If such a country or parts of the country did not have As-free water (Figs 2 and 3), then a 50 μg/L standard based on “treatment performance” could be lowered to a more protective <5 μg/L standard through treatment (Table 3). This would lower the lifetime cancer risk from 12,000 excess cancer deaths in 1,000,000 people (1 excess cancer death in 81 people) to <1,200 excess cancer deaths in 1,000,000 people (<1 excess cancer death in 810 people; see Table 3).

Countries with greater economic resources have access to more expensive instruments for testing and relatively sophisticated systems for treating drinking water. If such a country or parts of the country had As-free water (Figs 2 and 3), then a 10 μg/L standard based on analytical achievability could be lowered to a more protective ≤0.004 μg/L standard through testing and selective water use (Table 4). This would lower the lifetime cancer risk from 2,500 excess cancer deaths in 1,000,000 people (1 excess cancer death in 400 people) to ≤1 excess cancer deaths in 1,000,000 people (Table 4). If such country or parts of the country did not have As-free water (Figs 2 and 3), then a 10 μg/L standard based on treatment performance to a more protective 0.5 μg/L standard through more complete As treatment (Table 5). This would lower the lifetime cancer risk from 2,500 excess cancer deaths in 1,000,000 people (1 excess cancer death in 400 people) to 120 excess cancer deaths in 1,000,000 people (1 excess cancer death in 8,100 people; see Table 5).

## Supporting information

S1 File Arsenic regulations, populations, GDPs, and reference links for 195 countries. (XLSX)

## References

[1] Ahmed KM, Bhattacharya P, Hasan MA, Akhter SH, Alam SMM, Bhuyian MAH, et al. Arsenic enrichment in groundwater of the alluvial aquifers in Bangladesh: an overview. J Appl Geochem. 2004; 19:181–200. doi: 10.1016/j.apgeochem.2003.09.006

[2] Ali W, Rasool A, Junaid M, Zhang H. A comprehensive review on current status, mechanism and possible sources of arsenic contamination in groundwater: a global perspective with prominence of Pakistan scenario. Environ Geochem Health. 2019; 41-737–760. doi: 10.1007/s10653-018-0169-x 30101397

[3] Bhattacharya P, Welch AH, Ahmed KM, Jacks G, Naidu R. Arsenic in groundwater of sedimentary aquifers. Appl Geochem. 2004; 19:163–167. doi: 10.1016/j.apgeochem.2003.09.004

[4] Frisbie SH, Ortega R, Maynard DM, Sarkar B. The concentrations of arsenic and other toxic elements in Bangladesh’s drinking water. Environ Health Perspect. 2002; 110:1147–1153. doi: 10.1289/ehp.021101147 12417487 PMC1241072

[5] Jha SK, Mishra VK, Damodaran T, Sharma DK, Kumar P. Arsenic in the groundwater: Occurrence, toxicological activities, and remedies. J Environ Sci Health C. 2017; 35:84–103. doi: 10.1080/10590501.2017.1298359 28418774

[6] Mitchell EJ, Frisbie SH, Sarkar. Exposure to multiple metals from groundwater—a global crisis: Geology, climate change, health effects, testing, and mitigation. Metallomics. 2011; 3:874–908. doi: 10.1039/c1mt00052g 21766119

[7] Brown JP. Risk assessment for arsenic in drinking water. In: Howd RA, Fan AM, editors, Risk assessment for chemicals in drinking water. Hoboken: John Wiley & Sons, Inc; 2008, pp. 248–251.

[8] California Environmental Protection Agency (CalEPA). Public health goals for chemicals in drinking water. Arsenic. 2004. Available from: https://oehha.ca.gov/media/downloads/water/public-health-goal/asfinal.pdf. Cited 6 August 2017.

[9] Hasanvand M, Mohammadi R, Khoshnamvand N, Jafari A, Palangi HS, Mokhayeri Y. Dose-response meta-analysis of arsenic exposure in drinking water and intelligence quotient. J. Environ. Health Sci. Eng. 2020; 18:1691–1697. doi: 10.1007/s40201-020-00570-0 33312671 PMC7721833

[10] Kuo C-C, Moon KA, Wang S-L, Silbergeld E, Navas-Acien A. The association of arsenic metabolism with cancer, cardiovascular disease, and diabetes: a systematic review of the epidemiological evidence. Environ Health Perspect. 2017; 87:1–15. doi: 10.1289/EHP577 28796632 PMC5880251

[11] Mayer JE, Goldman RH. Arsenic and skin cancer in the USA: the current evidence regarding arsenic-contaminated drinking water. Int J Dermatol. 2016; 55:e585–e591. doi: 10.1111/ijd.13318 27420023

[12] Shakoor MB, Nawaz R, Hussain F, Raza M, Ali S, Rizwan M et al. Human health implications, risk assessment and remediation of As-contaminated water: a critical review. Sci. Total Environ. 2017; 601–602:756–769. doi: 10.1016/j.scitotenv.2017.05.223 28577410

[13] Sinha D, Prasad P. Health effects inflicted by chronic low-level arsenic contamination in groundwater: a global public health challenge. J Appl Toxicol. 2019; 40:87–131. doi: 10.1002/jat.3823 31273810

[14] Murcott S. Arsenic contamination in the world. An international sourcebook. London: IWA Publishing; 2012. doi: 10.1002/jsfa.4663

[15] Frisbie SH, Mitchell EJ, Roudeau S, Domart F, Carmona A, Ortega R. Manganese levels in infant formula and young child nutritional beverages in the United States and France: Comparison to breast milk and regulations. PLoS ONE. 2019; 14. doi: 10.1371/journal.pone.0223636 31689314 PMC6830775

[16] World Health Organization. International standards for drinking-water. 2nd edition. Geneva: World Health Organization; 1963. Available from: https://apps.who.int/iris/bitstream/handle/10665/205104/205104_eng.pdf;jsessionid = 2A6325A605F482131CAA0DAB8C9D35F6?sequence = 2. Cited 30 October, 2021.

[17] World Health Organization. Guidelines for drinking-water quality. Volume 1: Recommendations. 2nd ed. Geneva: World Health Organization; 1993. Available from: https://www.yumpu.com/en/document/view/32267650/guidelines-for-drinking-water-quality-volume-1-bvsde. Cited 30 October, 2021.

[18] World Health Organization. Guidelines for drinking-water quality. Fourth edition incorporating the first addendum. Geneva: World Health Organization; 2017. Available from: https://apps.who.int/iris/bitstream/handle/10665/254637/9789241549950-eng.pdf. Cited 30 October, 2021. 28759192

[19] United Nations. Member States. 2021. Available from: https://www.un.org/en/about-us/member-states. Cited 7 April 2021.

[20] The World Bank Group. GDP per capita. 2021. Available from: http://data.worldbank.org/indicator/NY.GDP.PCAP.CD. Cited 15 April 2021.

[21] Food and Agriculture Organization of the United Nations. FAOLEX Database. 2021. Available from: https://www.fao.org/faolex/en/. Cited 13 October 2021.

[22] Google Scholar. 2021. Available from: https://scholar.google.com/. Cited 13 October 2021.

[23] Google Translate. 2021. Available from: https://translate.google.com. Cited 13 October 2021.

[24] World Health Organization. A global overview of national regulations and standards for drinking-water quality. 2018. Available from: https://apps.who.int/iris/rest/bitstreams/1135599/retrieve. Cited 10 September 2021.

[25] International Water Resources Association. Developing a global compendium on water quality guidelines. Paris: International Water Resources Association; 2018. Available from: https://www.iwra.org/wp-content/uploads/2018/11/WQ-compendium-final-1.pdf. Cited 13 July 2021.

[26] Slavik I, Oliveira KR, Cheung PB, Uhl W. Water quality aspects related to domestic drinking water storage tanks and consideration in current standards and guidelines throughout the world–a review. Journal of Water and Health. 2020; 18:439–463. doi: 10.2166/wh.2020.052 32833673

[27] Truque PA. Armonizacion de los estandares de agua potable en las Americas. [2012]. Available from: https://www.oas.org/dsd/publications/classifications/Armoniz.EstandaresAguaPotable.pdf. Cited 26 April 2021.

[28] The World Bank Group. Population. 2021. Available from: http://data.worldbank.org/indicator/ SP.POP.TOTL. Cited 15 April 2021.

[29] The World Bank Group. GDP. Available from: http://data.worldbank.org/indicator/ NY.GDP.MKTP.CD/1ff4a498/Popular-Indicators#. Cited 15 April 2021.

[30] The World Bank Group. Global economic prospects, January 2020: slow growth, policy changes. 2020. Available from: https://elibrary.worldbank.org/doi/pdf/10.1596/978-1-4648-1469-3. Cited 15 April 2021.

[31] The World Bank Group. The World Bank list of economies. 2021. Available from: https://databank.worldbank.org/data/download/site-content/CLASS.xls. Cited 15 April 2021.

[32] [People’s Democratic Republic of Algeria]. Décret exécutif n° 11–125 du 17 Rabie Ethani 1432 correspondant au 22 mars 2011 relatif à la qualité de l’eau de consommation humaine. Journal Officiel de la Republique Algerienne. 23 March 2011; n° 18. Available from: http://extwprlegs1.fao.org/docs/pdf/alg106153.pdf. Cited 4 June 2021.

[33] [Republic of Angola]. Regulamento sobre a qualidade da água. Diário da república. 6 October 2011; I series—no 193. Available from: http://faolex.fao.org/docs/pdf/ang119447.pdf. Cited 4 June 2021.

[34] [Republic of Benin]. Decret no 2001–094 du 20 fevrier 2001 fixant les norms de qualite de l’eau potable en Republique du Benin. Porto-Novo; 20 February 2001. Available from: http://faolex.fao.org/docs/pdf/ben86060.pdf. Cited 4 June 4 2021].

[35] Botswana Bureau of Standards. Water quality: drinking water. BOS 32:2009. Gabarone: Botswana Bureau of Standards. 2009. [Not obtained. May be available for purchase in person in Gabarone].

[36] Botswana Bureau of Standards. Botswana Standards. Standards Catalogue. Search facilities. 2021. Available from: http://www.bobstandards.bw/Pages/Search-Facilities.aspx?mnusub=33&pid=30&mp=0&sp=18. Cited 13 October 2021.

[37] Centre for Applied Research. Draft Botswana country water report. Prepared for UN ECA as part of the preparation of the African water Development Report. Addis Ababa: Centre for Applied Research; March 2005. Available from: https://www.car.org.bw/wp-content/uploads/2016/06/Botswana-water-report-UNECA-draft-final-subm.pdf. Cited 5 June 2021.

[38] [Popular Democratic Republic of Burkina Faso]. Directives de qualité pour l’eau de boisson. 2nd edition. Arrêté conjoint n° 0019/MAHRH/MS du 05 avril 2005. Jo. 19 May 2005; 20. Available from: http://extwprlegs1.fao.org/docs/texts/bkf53276.doc. Cited 4 June 4 2021.

[39] Republique du Burundi. Ministere du Commerce, de l’industrie des postes et du tourisme. Cabinet du Ministre. No Réf: 750/1028/C.M./2014. Annonce. Bujumbura: Ministere du Commerce, de l’industrie des postes et du tourisme; 2014. Available from: http://bbn-burundi.org/Documents/normes_obligatoires.pdf. Cited 26 March 2018.

[40] East African Standards Committee. Potable Water—Specification. East African Draft Standard. EAS 12:2000. Arusha, Tanzania: East African Standards Committee; 2000. Available from: https://silo.tips/download/draft-for-comments-only-not-to-be-cited-as-east-african-standard. Cited 13 October 2021.

[41] Mananga M-J, Sop MMK, Nguidjol E, Gouado I. Quality of packaged drinking water marketed in Douala—Cameroon. J Water Res Ocean Sci. 2014; 3: 74–79. doi: 10.11648/j.wros.20140306.12

[42] [Republic of Cabo Verde]. Decreto-Lei n.o 8/2004 de 23 de Fevereiro. B.O. da República de Cabo Verde. 23 February 2004; I série—no 6. Available from: http://extwprlegs1.fao.org/docs/pdf/cvi46956.pdf. Cited 6 June 2021.

[43] Republique Centrafricaine. Loi n° 06.001 du 12 avril 2006 portant code de l’eau de la Republique Centrafricaine. Bangui; 12 April 2006. Available from: http://extwprlegs1.fao.org/docs/pdf/caf107433.pdf. Cited 6 June 2021.

[44] République du Tchad. Ministere de l’Eau. Decret no. 15/PR/PM/ME/MSP/2010 portant définition nationale de l’eau potable au Tchad. N’Djamena: Ministere de l’Eau; 31 March 2010. Available from: http://extwprlegs1.fao.org/docs/pdf/cha147100.pdf. Cited 6 June 2021.

[45] [Federal Islamic Republic of Comoros]. Loi n° 94–037 du 21 Décembre 1994 portant code de l’eau. Moroni; 21 December 1994. Available from: http://faolex.fao.org/docs/texts/com78264.doc. Cited 12 June 2018.

[46] [Republic of Djibouti]. Décret n° 2001-0010/PR/MCIA réglementation des eaux conditionnées destinées à la consommation humaine. Djibouti; 9 January 2001. Available from: http://faolex.fao.org/docs/texts/dji38341.doc. Cited 8 June 2021.

[47] [Arab Republic of Egypt]. Minister of Health decree number (458) for 2007. [Not obtained.]

[48] Elewa AMT, El Sayed E, El Kashouty M, Morsi M. Quantitative study of surface and groundwater systems in the western part of the River Nile, Minia Governorate, Upper Egypt: water quality in relation to anthropogenic activities. Greener J Phys Sci. 2013; 3: 212–228.

[49] Ethiopian Standards Agency. Drinking water—specifications. CES 58. 2013. Addis Ababa: Ethiopian Standards Agency; 2013. Available from: https://www.humanitarianresponse.info/sites/www.humanitarianresponse.info/files/documents/files/drinking_water_specifications.pdf. Cited 9 June 2021.

[50] République Gabonaise. Ministere de la Sante, des Affaires Sociales, de la Solidarite et et la Famille. Normes du secteur de la santé. Libreville: Ministere de la Sante, des Affaires Sociales, de la Solidarite et et la Famille; January 2011. Available from: http://csgabon.info/file/f2/Normessante%2014072011.pdf. Cited 10 June 2021.

[51] Ghana Standards Authority. Water quality- specification for drinking water. Ed. 5. GS 175:2017. Accra: Ghana Standards Authority; 2017. [Not obtained. Available for purchase in person in Accra.]

[52] Ghana Standards Authority. Catalogue of Ghana standards. 2018. Available from: https://www.gsa.gov.gh/wp-content/uploads/2018/08/2018-Catalogue-of-Ghana-Standards.pdf. Cited 10 June 2021.

[53] [Government of Ghana]. Ministry of Water Resources, Works and Housing. National drinking water quality management framework for Ghana. Accra: Ministry of Water Resources, Works and Housing; June 2015. Available from: http://www.gwcl.com.gh/national_drinking_water_quality__management_framework.pdf. Cited 10 June 2021.

[54] République de Guinée. Code de la sante publique. Conakry; 19 June 1997. Available from: https://sites.google.com/site/guineejuristes/CSANTEPUBLIQUE.pdf. Cited 11 June 2021.

[55] Republique de Cote d’Ivoire. Loi n°98–755 du 23 décembre 1998 portant code de l’eau. Yamoussoukro; 23 December 1998. Available from: http://extwprlegs1.fao.org/docs/pdf/ivc15630.pdf. Cited 12 June 2021.

[56] Kenya Bureau of Standards. Potable water—specification. KS EAS 12: 2018. Nairobi: Kenya Bureau of Standards; 2018. [Not obtained. Available for purchase online from https://webstore.kebs.org/.]

[57] Kenya Bureau of Standards. Web catalogue access. 2021. Available from: http://onlinecatalogue.kebs.org/webquery.dll?v1=pbMarc&v4=0&v5=5D&v8=442602&v9=1&v10=N&v13=4D&v20=4&v22=4D@KS%201923:2007&v23=0&v25=POTABLE%20and%20%20WATER&v27=14909&v29=5D&v35={]0[}{]0[}{]0[}{]0[}&v40=442600&v46=442602. Cited 5 May 2021.

[58] Kenya Bureau of Standards. Potable water—specification. KS EAS 12: 2014. Nairobi: Kenya Bureau of Standards; 2014. Available from: http://onlinecatalogue.kebs.org/webquery.dll?v1=pbMarc&v4=0&v5=5D&v8=442602&v9=1&v10=N&v13=4D&v20=4&v22=4D@KS%201923:2007&v23=0&v25=POTABLE%20and%20WATER&v27=14909&v29=5D&v35={]0[}{]0[}{]0[}{]0[}&v40=442600&v46=442602. Cited 14 June 2021.

[59] Heller L. Statement at the conclusion of the official visit to Lesotho by the Special Rapporteur on the human rights to safe drinking water and sanitation. Geneva: United Nations Human Rights Special Procedures. Special Rapporteurs, Independent Experts & Working Groups; 15 February 2019. Available from: https://www.ohchr.org/Documents/Issues/Water/EndofMissionLesotho.pdf. Cited 6 May 2021.

[60] The Republic of Liberia. Ministry of Lands, Mines and Energy. National integrated water resources management policy. Monrovia: Ministry of Lands, Mines and Energy; November 2007. Available from: http://extwprlegs1.fao.org/docs/pdf/lbr180020.pdf. Cited 6 May 2021.

[61] Libyan National Center of Standardization and Metrology. [Drinking water standard]. Tripoli: Libyan National Center of Standardization and Metrology; 2015. [Not obtained. Available for purchase in person in Tripoli.]

[62] Libyan National Center of Standardization and Metrology. Personal communication. 2021 May 9.

[63] Taher SE-DE-L. Quality evaluation of drinking water sources in Al-Gabal Al-Akhdar Region, Libya. Arab J Nucl Sci Appl. 2016; 49: 26–30.

[64] [Republic of Madagascar]. Norme de potabilite malagasy (décret n°2004–635 du 15/06/04). Antananarivo; 15 June,2004. Available from: https://www.jirama.mg/wp-content/uploads/2020/05/NORME-DE-POTABILITE-MALAGASY-De%cc%81cret-n2004-635_du_2015.06.04-1.pdf. Cited 31 May 2021.

[65] Malawi Bureau of Standards. Drinking water—specification. 2^nd^ edition. MS 214:2013. Blantyre: Malawi Bureau of Standards; 2013. [Not obtained. Available for purchase in person in Blantyre.]

[66] Malawi Bureau of Standards. Catalogue of Malawi standards. 2019. Available from: http://mbsmw.org/wp-content/uploads/2019/05/2019-Catalogue-of-Malawi-Standards.pdf. Cited 14 October 2021.

[67] Ngumbira KS. An analysis of the physico-chemical and microbial quality of sachet water in Lilongwe, Malawi: Implication on public health and WASH policies. M.Sc. Thesis. Lagos, Nigeria: Pan-African University; 2020. Available from: http://repository.pauwes-cop.net/bitstream/handle/1/409/Master Thesis Kizito_ Final_endorsed.pdf. Cited 28 June 2021.

[68] République du Mali. Ministere de l’energie, des mines et de l’eau. Stratégie nationale de developpement de l’alimentation en eau potable au Mali. Bamako: Ministere de l’energie, des mines et de l’eau; 28 November 2007. Available from: https://www.dnhmali.org/IMG/Strategie_AEPA.pdf. Cited 31 May 2021.

[69] Republique Islamique de Mauritanie. Ministere de L’hydraulique et de L’assainissement. Direction de l’hydraulique. Normes pour l’alimentation en eau potable en milieu rural et semiurbain. Nouakchott: Ministere de L’hydraulique et de L’assainissement; May 2015. Available from: https://www.pseau.org/outils/ouvrages/mha_normes_et_guide_pour_l_alimentation_en_eau_potable_en_milieu_rural_et_semi_urbain_vol1_2015.pdf. Cited 23 June 2021.

[70] [Republic of Mauritius.] Drinking water standards. Legal supplement to the Government Gazette of Mauritius no. 72 of 22 June 1996. Government notice no. 55 of 1996. The Environment Protection Act 1991. Government Gazette of Mauritius. 22 June, 1996; 72 supplement. Available from: http://extwprlegs1.fao.org/docs/texts/mat52516.doc. Cited 23 June 2021.

[71] Institute Marocain de Normalisation. Qualité des eaux à usage alimentaire. NM 03.7.001. Rabat: Institute Marocain de Normalisation; 2020. [Not obtained. Available for purchase in person in Rabat.]

[72] Institute Marocain de Normalisation. Recherche d’une norme et catalogue. Available from https://www.imanor.gov.ma/normes/page/39/. Cited 15 October 2021.

[73] Service de Normalisation Industrielle Marocaine. Qualite des eaux d’alimentation humaine. Norme Marocaine NM 03.7.001. Rabat: Service de Normalisation Industrielle Marocaine; [2006]. Available from: https://pdfcoffee.com/nm-037001-norme-maroccaine-eau-alimentation-4-pdf-free.html. Cited 2 June 2021.

[74] [Republic of Mozambique]. Regulamento sobre a qualidade da agua para o consumo humano. Boletim da Republica. Diploma ministerial no. 180/2004 [Regulation on the quality of water for human consumption. Boletim da Republica. 15 September 2004; Ministerial diploma no. 180/2004]. Available from: http://extwprlegs1.fao.org/docs/pdf/moz65565.pdf. Cited 26 June 2021.

[75] [Republic of Namibia]. [Ministry of Agriculture, Water and Rural Development. Department of Water Affairs]. The water act (act 54 of 1956) and its requirements in terms of water supplies for drinking water and for waste water treatment and discharge. Windhoek: Ministry of Agriculture, Water and Rural Development; [1988]. Available from: http://www.envirod.com/enviro_admin/assets/documents/p1946kkpio1d221aklron1rt711d0d.pdf. Cited 27 June 2021.

[76] Kempster PL, Smith R. Proposed aesthetic/physical and inorganic drinking-water criteria for the Republic of South Africa. CSIR Research Report No. 628. Pretoria: National Institute for Water Research; 1985. Available from: https://researchspace.csir.co.za/dspace/bitstream/handle/10204/7312/Smith_1985.pdf?sequence=1&isAllowed=y. Cited 13 July 2021.

[77] République du Niger. Presidence de la Republique. Ministere de l’Hydraulique et de l’Environnement. Décret n° 97-368/PRN/MH/E déterminant les modalités d’application de l’ordonnance n° 93–014 du 2 Mars 1993 portant régime de l’eau. Niamey: Ministere de l’Hydraulique et de l’Environnement; 2 October 1997. Available from: http://extwprlegs1.fao.org/docs/pdf/ner13579.pdf. Cited 29 June 2021.

[78] Standards Organisation of Nigeria. Nigerian standard for drinking water quality. Nigerian industrial standard NIS-554-2015. Abuja: Standards Organisation of Nigeria; 2015. Available from: https://rivwamis.riversstate.gov.ng/assets/files/Nigerian-Standard-for-Drinking-Water-Quality-NIS-554-2015.pdf. Cited 29 June 2021.

[79] Rwanda Standards Board. Potable water—specification. 2nd edition. RSB 2014-11-28. Kigali: Rwanda Standards Board; 2014. [Not obtained. Available for purchase in person in Kigali.]

[80] Republic of Rwanda. Ministry of Infrastructure. Rural drinking water quality management framework. Kigali: Ministry of Infrastructure; May 2019. Available from: https://www.mininfra.gov.rw/fileadmin/user_upload/Mininfra/Documents/Water_and_Sanitation_docs/2_Rural_Drinking_Water_Quality_Framework.pdf. Cited 6 July 2021.

[81] Vidal J. Water and sanitation still not top priorities for African Governments. The Guardian. 30 August 2012. Available from: https://www.theguardian.com/global-development/2012/aug/30/water-sanitation-priorities-african-governments. Cited 14 July 2021.

[82] République du Sénégal. Ministerie de l’Hydraulique et de l’Assainissement. Cadre de gestion environmentale et sociale du projet eau et assainissement en milieu rural (PEAMIR). Dakar: Ministerie de l’Hydraulique et de l’Assainissement; March 2018. Available from: https://documents1.worldbank.org/curated/en/286951523475775149/pdf/Cadre-de-gestion-environmentale-et-sociale.pdf. Cited 12 July 2021.

[83] [Republic of Seychelles]. Public health (water examination) regulations. Victoria; 30 June 2012. Available from: http://extwprlegs1.fao.org/docs/pdf/sey139464.pdf. Cited 14 July 2021.

[84] The Sierra Leone Electricity and Water Regulatory Commission. The water (quality of supply) regulations 2019 (act no 13 of 2011). Statutory Instrument No. 20 of 2019. Freetown: The Sierra Leone Electricity and Water Regulatory Commission; 2019. Available from: http://extwprlegs1.fao.org/docs/pdf/sie203325.pdf. Cited 14 July 2021.

[85] South African Bureau of Standards. South African National Standard. Drinking water. Part 1: Microbiological, physical, aesthetic and chemical determinands. Edition 2. SANS 241–1:2015. Pretoria: South African Bureau of Standards; 2015. Available from: https://store.sabs.co.za/pdfpreview.php?hash=d3d0b4e624a31e2a7a68cf1a3f4fb181b864dcdf&preview=yes. Cited 14 July 2021.

[86] South African Bureau of Standards. South African National Standard. Drinking water. SANS 241–1:2015. Pretoria: South African Bureau of Standards; 2015. Available from: https://www.mwa.co.th/download/prd01/iDW_standard/South_African_Water_Standard_SANS_241-2015.pdf. Cited 14 July 2021.

[87] United Nations International Children’s Emergency Fund. Southern Sudan water quality guidelines. Juba: UNICEF; October 2008. Available from: https://www.yumpu.com/en/document/read/37070914/southern-sudan-water-quality-guidelines-basic-services-fund-. Cited 15 July 2021.

[88] Sudanese Standards and Metrology Organization. Drinking water standard, ICS 13.060.00. Khartoum: Sudanese Standards and Metrology Organization; 2006. [Not obtained. May be available for purchase in person in Khartoum.]

[89] Deen ASI. Groundwater quality assessment in EL Fasher area. M.Sc. Thesis. Khartoum: Al Neelain University; 2017. Available from: http://repository.neelain.edu.sd:8080/jspui/bitstream/123456789/12470/1/Abstract.pdf. Cited 5 September 2018.

[90] Eswatini Standards Authority. Drinking water. SZNS SANS 241–1: 2015. Matsapha: Eswatini Standards Authority; 2015. [Not obtained. Available for purchase online at https://shop.swasa.online].

[91] Eswatini Standards Authority. Standards Catalog. Matsapha: Eswatini Standards Authority; 2021. Available from: https://shop.swasa.online/catalogue/. Cited 18 October 2021.

[92] Tanzania Bureau of Standards. Portable water specification. TZS789:2018-EAS12:2018. Dar es Salaam: Tanzania Bureau of Standards; 2018. [Not obtained. Available for purchase online at https://standards.tbs.go.tz/].

[93] Tanzania Bureau of Standards. Standard Catalogues. 2021. Available from: https://tbs.go.tz/pages/standards-catalogue. Cited 18 October 2021.

[94] Energy and Water Utilities Regulatory Authority. Water and wastewater quality monitoring guidelines for water supply and sanitation authorities. 2nd ed. Dar es Salaam: Energy and Water Utilities Regulatory Authority; March 2020. Available from: https://www.ewura.go.tz/wp-content/uploads/2020/06/Water-and-Wastewater-Quality-Monitoring-Guidelines-2020.pdf. Cited 31 July 2021.

[95] Public Utilities Regulatory Authority (PURA). Common guidelines on minimum quality of service standards for water and sanitation. Banjul: Public Utilities Regulatory Authority; November 2008. Available from: http://extwprlegs1.fao.org/docs/pdf/gam188566.pdf. Cited 12 July 2021.

[96] République Togolaise. Ministere de l’Agriculture, de l’Elevage et de l’Hydraulique. Projet de normes Togolaises de qualite pour l’eau de boisson. Lomé: Ministere de l’Agriculture, de l’Elevage et de l’Hydraulique; December 2015. Available from: https://www.pseau.org/outils/ouvrages/projet_de_normes_togolaises_de_qualite_pour_l_eau_de_boisson_2015.pdf. Cited 31 July 2021.

[97] Institut National de la Normalisation et de la Propriété Industrielle. Eaux destinées à la consommation humaine à l’exclusion des eaux conditionnées. Tunis: Institut National de la Normalisation et de la Propriété Industrielle; 4 April 2013. Available from: http://www.innorpi.tn:8080/web/guest/normes?p_p_id=EXT_2&p_p_action=0&p_p_state=maximized&p_p_mode=view&p_p_col_id=&p_p_col_pos=0&p_p_col_count=0&_EXT_2_struts_action=%2Fext%2Fcatalogue_normes%2Fview&_EXT_2_tabs1=normes-tunisiennes&_EXT_2_group=&group=&normetn=NT%2009.14(2013)&normeet=&tabs1=normes-tunisiennes. Cited 3 August 2021.

[98] Uganda National Bureau of Standards. Potable Water—Specification. US EAS 12. 2014 Kampala: Uganda National Bureau of Standards; 15 October 2014. Available from: https://members.wto.org/crnattachments/2015/TBT/UGA/15_0152_00_e.pdf. Cited 24 August 2021.

[99] Zambia Bureau of Standards. Drinking water quality—specification. Zambian Standard (first revision). ZS 190: 2010. Lusaka: Zambia Bureau of Standards; 23 February 2010. Available from: http://www.puntofocal.gov.ar/notific_otros_miembros/zmb48_t.pdf. Cited 31 August 2021.

[100] Standards Association of Zimbabwe. Natural mineral water and spring water. ZWS 457: 2015. Harare: Standards Association of Zimbabwe; 2015. [Not obtained. Available for purchase online from: https://saz.org.zw/].

[101] Standards Association of Zimbabwe. Water for domestic supplies. ZWS 560: 2004. Reprinted 2004. Harare: Standards Association of Zimbabwe; 2004. [Not obtained. Available for purchase online from: https://saz.org.zw/].

[102] Standards Association of Zimbabwe. Packaged drinking water other than natural mineral water. ZWS 791: 2015. Harare: Standards Association of Zimbabwe; 2015. [Not obtained. Available for purchase online from: https://saz.org.zw/].

[103] Standards Association of Zimbabwe. Draft SAZ specification for packaged drinking water other than natural mineral water. Draft number FD 009-D 791/1. Harare: Standards Association of Zimbabwe; 2015. Available from: https://www.coursehero.com/file/16241491/An-Example-of-a-SAZ-Water-Standard/. Cited 18 October 2021.

[104] Munsaka P. A quantitative analysis of Kamativi’s water quality. Master’s thesis. Christchurch, New Zealand: University of Canterbury; 2017. Available from: https://ekgotla.files.wordpress.com/2015/09/zimbabwe-municipalities-proposed-prepaid-water-bid-for-impact-study-docx.pdf. Cited 26 August 2021.

[105] [Government of Antigua and Barbuda]. Ministerio de Sanidad y Consumo. Royal decree 140/2003 of 7 February by which health criteria for the quality of water intended for human consumption are established. 2003 February 7. Available from: https://www.mscbs.gob.es/profesionales/saludPublica/docs/royal_decree_140_2003.pdf. Cited 5 June 2021.

[106] CARICOM Regional Organisation for Standards & Quality. CARICOM Regional Standard. Specification for packaged water. CRS 1:2010. Bridgetown: CARICOM Regional Organisation for Standards & Quality; 2010. Available from https://law.resource.org/pub/crs/ibr/cc.crs.1.2010.html. Cited 18 October 2021.

[107] [Argentine Republic]. [Código alimentario Argentino]. Capítulo XII. Bebidas hídricas, agua y agua gasificada. Agua potable, Artículo 982. Resolución conjunta SRYGR y SAB n° 34/2019. Buenos Aires; 2019. Available from: https://www.argentina.gob.ar/sites/default/files/caa_capitulo_xii_aguas_actualiz_2021-01.pdf. Cited 20 April 2021.

[108] The Bahamas Bureau of Standards. Draft Bahamas national standard specification for packaged water. DBNSS 1:2014 CRS 1:2010. Nassau: The Bahamas Bureau of Standards; 2010. Available from: https://www.bahamas.gov.bs/wps/wcm/connect/59655bcc-a295-437c-a941-04a6af9c4b46/DBNSS+1-+Specification+Packaged+Water+Jan+21+2015.pdf?MOD=AJPERES. Cited 5 June 2021.

[109] Barbados Fair Trading Commission. Decision–Barbados Water Authority standards of service 2018–2020. Bridgetown: Barbados Fair Trading Commission; 31 May 2017. Available from: https://www.ftc.gov.bb/library/sos/2017-05-31_commission_decision_sos_bwa_2018-2020.pdf. Cited 5 June 2021.

[110] Amandala Newspaper. Belize water analyst says there is need for continued monitoring. 20 October 2009. Available from: https://amandala.com.bz/news/belize-water-analyst-says-there-is-need-for-continued-monitoring/. Cited 5 June 2021.

[111] Instituto Boliviano de Normalización y Calidad. Compendio normativo sobre calidad del agua para consumo humano. NB 512 –Reglamento NB 512 –NB 495 –NB 496. La Paz: Instituto Boliviano de Normalización y Calidad; October 2018. Available from: https://www.bivica.org/files/normativa-calidad-agua.pdf. Cited 5 June 2021.

[112] [Federative Republic of Brazil]. Portaria GM/MS No 888, de 4 de Maio de 2021. Anex XX à Portaria de Consolidação n° 5/GM/MS, de 28 de setembro de 2017. Procedimentos de controle e de vigilância da qualidade da água para consumo humano e seu padrão de potabilidade. Diáfio official da uniã. 4 May 2021; 85(1): 127. Available from: https://www.in.gov.br/web/dou/-/portaria-gm/ms-n-888-de-4-de-maio-de-2021-318461562. Cited 22 April 2021.

[113] Health Canada. Guidelines for Canadian drinking water quality. Summary table. Ottowa: Health Canada; September 2020. Available from: https://www.canada.ca/content/dam/hc-sc/migration/hc-sc/ewh-semt/alt_formats/pdf/pubs/water-eau/sum_guide-res_recom/summary-table-EN-2020-02-11.pdf. Cited 6 June 2021.

[114] [Republic of Chile]. Modifica el decreto n° 735, de 1969, reglamento de los servicios de agua destinados al consumo humano. Santiago; 26 March 2007. Available from: https://www.leychile.cl/Navegar?idNorma=259363. Cited 6 June 2021.

[115] [Republic of Colombia]. Resolución conjunta 2115 de 2007 por medio de la cual se señalan características, instrumentos básicos y frecuencias del sistema de control y vigilancia para la calidad del agua para consumo humano. Diario Oficial. 4 July 2007; 46679. Available from: http://www.alcaldiabogota.gov.co/sisjur/normas/Norma1.jsp?i=30008. Cited 7 June 2021.

[116] [Republic of Costa Rica]. Decreto 38924-S. Reglamento para la calidad del agua potable. La Gaceta. 1 September, 2015; 170. Available from: https://www.aya.go.cr/laboratorio/selloCalidad/requisitosGalardon/Decreto Ejecutivo No 38924-S. Reglamento para la calidad del agua potable.pdf. Cited 7 June 2021.

[117] Oficina Nacional de Normalización. Agua potable—requisitos sanitarios. NC 827: 2017. La Habana: Oficina Nacional de Normalización; December 2017. Available from: http://www.cgdc.cu/sites/default/files/nc_827.pdf. Cited 7 June 2021.

[118] Economic Commission for Latin America and the Caribbean (ECLAC). Subregional Headquarters for the Caribbean. Overview of the Water Profile and the Capacity of National Institutions to Implement Integrated Water Resources Management (Antigua and Barbuda, Dominica, Grenada). Santiago, Chile: Economic Commission for Latin America and the Caribbean; 24 November 2007. Available from: https://www.cepal.org/sites/default/files/publication/files/27629/LCcarL143_en.pdf. Cited 8 June 2021.

[119] [Dominican Republic]. Secretaria de Estado de Medio Ambiente y Recursos Naturales. Norma de calidad del agua y control de descargas, AG-CC-O1. Santo Domingo: Secretaria de Estado de Medio Ambiente y Recursos Naturales; 2001. Available from: http://www.fao.org/faolex/results/details/en/c/LEX-FAOC060779. Cited June 2021.

[120] [Republic of Ecuador]. Norma de calidad ambiental y de descarga de efluentes: Recurso agua. Anexo 1 del libro vi del texto unificado de legislacion secundaria del Ministerio del Ambiente. Registro official; 4 November 2015; edición especial n° 387. Available from: http://extwprlegs1.fao.org/docs/pdf/ecu155128.pdf. Cited June 2021.

[121] [Republic of El Salvador]. Ministerio de Salud. Agua, agua potable. Segunda actualización. Norma Salvadoreña NSO 13.07.01:08. Diario Oficial. 12 June 2009; 383(109). Available from: http://usam.salud.gob.sv/archivos/pdf/normas/NORMA_AGUA_POTABLE_2_a.pdf. Cited 9 June 2021.

[122] [Government of Grenada]. Act No. 1 of 2005. Chapter 334B. Water quality act. An Act to govern matters relating to the Quality of Water Intended for Human Consumption. Saint George’s; 7 January 2005. Available from: http://extwprlegs1.fao.org/docs/pdf/grn180623.pdf. Cited 11 June 2021.

[123] World Health Organization. Guidelines for drinking-water quality. Volume 1: Recommendations. 3rd ed. Geneva: WHO; 2004. Available from: https://www.who.int/water_sanitation_health/dwq/GDWQ2004web.pdf. Cited 30 October, 2021.

[124] Comisión Guatemalteca de Normas. Agua para consumo humano (agua potable). Especificaciones. Referencia ICS: 13.060.20. Guatemala City: Comisión Guatemalteca de Normas; 2013. Available from: https://www.mspas.gob.gt/images/files/saludabmiente/regulacionesvigentes/AguaConsumoHumano/NormaTecnicaGuatemaltecaNTG29001.pdf. Cited 11 June 2021.

[125] République d’Haïti. Direction Nationale de l’Eau Potable et de l’Assainissement. Directive technique. Procédés de désinfection et postes de dosage. Port au Prince: Direction Nationale de l’Eau Potable et de l’Assainissement; 9 September 2013. Available from: https://dinepa.gouv.ht/referentieltechnique/doc/1-aep/1.2.2%20DIT1%20Procedes%20de%20desinfection%20et%20postes%20de%20dosage.pdf. Cited 11 June 2021.

[126] [Republic of Honduras]. Reglamento técnico de calidad de agua envasada y hielo para consume humano directo e indirecto. La Gaceta. 14 August 2007; 31.381. Available from: http://faolex.fao.org/docs/pdf/hon92610.pdf. Cited 11 June 2021.

[127] Government of Jamaica. Ministry of Economic Growth and Job Creation. National water sector policy and implementation plan 2019. Kingston: Ministry of Economic Growth and Job Creation; 2019. Available from: https://megjc.gov.jm/docs/policies/national_water_sector_policy_2019.pdf. Cited 13 June 2021.

[128] [United Mexican States]. Proyecto de Norma Oficial Mexicana PROY-NOM-127-SSA1-2017, agua para uso y consumo humano. Límites permisibles de la calidad del agua. Mexico City; 12 June 2019. Available from: https://www.dof.gob.mx/nota_detalle.php?codigo=5581179&fecha=06/12/2019. Cited 24 June 2021.

[129] [Republic of Nicaragua]. Norma tecnica obligatoria nicaraguense. Norma para la classification de los recursos hidricos. Reg. No. 10116 – M.039158. La Gaceta. 11 February 2000; 30:755–758. Available from: http://legislacion.asamblea.gob.ni/normaweb.nsf/($All)/1A3A99B77290B980062573DF00594022?OpenDocument. Cited 29 June 2021.

[130] [Republic of Panama]. Agua Envasada. Requistios generals. Gaceta Oficial Digital. 17 December 2007; No. 25941. Available from: https://slidex.tips/download/ministerio-de-la-presidencia-decreto-n-136-de-martes-6-de-noviembre-de-2007. Cited 1 July 2021.

[131] [Republic of Paraguay]. Ley general del marco regulatorio y tarifario del servicio de agua potable y alcantarillado sanitario. Ley n° 1.614/2000. Reglamento de calidad en la prestación del servicio permisionarios. Asunción; 2000. Available from: https://www.undp.org/content/dam/paraguay/docs/2-reglamento_de_calidad_para_permisionarios.pdf. Cited 2 July 2021.

[132] World Health Organization. International standards for drinking-water. 3rd ed. Geneva: WHO; 1971. Available from: https://apps.who.int/iris/bitstream/handle/10665/39989/9241540249_eng.pdf?sequence=1&isAllowed=y. Cited 30 October, 2021.

[133] [Republic of Peru]. Aprueban estándares de calidad ambiental (ECA) para agua y establecen disposiciones complementarias decreto. Supremo n° 004-2017-MINAM. El Peruano. 7 June 2017. Available from: http://extwprlegs1.fao.org/docs/pdf/per171691.pdf. Cited 2 July 2021.

[134] Smith G. Environmental and social analysis water supply modernization program SU-L1058. Fit for disclosure report. Paramaribo: Suriname Water Supply Modification Program; 30 October 2019. Available from: https://www.swm.sr/wp-content/uploads/2019/11/Fit-for-Disclosure-ESA-SU-L1058-1.pdf. Cited 23 July 2021.

[135] United States Environmental Protection Agency. 2018 Edition of the drinking water standards and health advisories tables. EPA 822-F-18-001. Washington, DC: U.S. Environmental Protection Agency; 2018. Available from: https://www.epa.gov/sites/production/files/2018-03/documents/dwtable2018.pdf. Cited 12 July 2021.

[136] Instituto Uruguayo de Normas Técnicas. Agua potable—Requisitos. Reimpresión corregida. UNIT 833:2008. Montevideo: Instituto Uruguayo de Normas Técnicas; July 2010. Available from: http://www.ose.com.uy/descargas/Clientes/Reglamentos/unit_833_2008_.pdf. Cited 25 August 2021.

[137] [Bolivarian Republic of Venezuela]. Normas sanitarias de calidad del agua potable. Gaceta Oficial de la Republica de Venezuela. 13 Febuary 1998; 303:216–218. Available from: http://www.cipram.com.ve/pdf/Normas%20Sanitarias%20de%20Calidad%20del%20Agua%20Potable.pdf. Cited 31 August 2021.

[138] Afghan National Standards Authority. Afghanistan national drinking water quality standards. ANSA/TC 14. AS..:2012. Kabul: Afghan National Standards Authority; 2013. [Provided 5 June 2021 by an anonymous contact at the Rural Water Supply & Sanitation Program, Ministry of Rural Rehabilitation & Development, Kabul].

[139] Republic of Armenia. On regulations for establishing water standards. Yerevan; 2005. [Not obtained].

[140] Republic of Armenia and the European Union Water Initiative. Revised national targets of Armenia in the context of the UNECE-WHO/Europe protocol on water and health. Brussels: European Union Water Initiative; August 2019. Available from: https://unece.org/fileadmin/DAM/env/water/Protocol_on_W_H/Target_set_other_states/Armenia/EUWI__Armenia_publication-final.pdf. Cited 12 July 2021.

[141] United Nations Economic Commission for Europe. Protocol on water and health–improving health in Armenia through target setting to ensure sustainable water management, access to safe water and adequate sanitation. Geneva: United Nations Economic Commission for Europe; 2014. Available from: https://www.unece.org/fileadmin/DAM/env/water/npd/Armenia/baseline-eng-final.pdf. Cited 16 April 2018.

[142] “Azersu” Open Joint Stock Company. Standards. Available from: https://azersu.az/en/static/8/link/7. Cited 18 October 2021.

[143] [Soviet Union]. Ипк Издательство Стандартов. Вода питьевая гигиенические требования и контроль за качеством. Гост 2874–82. Moscow: Ипк Издательство Стандартов; 1985. Available from: https://ohranatruda.ru/ot_biblio/norma/220583/. Cited 31 July 2021.

[144] Kingdom of Bahrain. Electricity and Water Authority. Water treatment review. Table 2.1: Comparison of water quality standards and recommendations for Bahrain. Available from: https://www.ewa.bh/en/Network/Water/Documents/WTD%20water%20standards.pdf. Cited 20 April 2021.

[145] GCC Standardization Organization (GSO). Unbottled drinking water. GSO5/DS/…/2012. Riyadh: GCC Standardization Organization (GSO); 2012. Available from: https://members.wto.org/crnattachments/2014/sps/OMN/14_2156_00_e.pdf. Cited 20 April 2021.

[146] [People’s Republic of Bangladesh]. Department of Public Health Engineering. Water quality parameters. Last updated: 15th May 2019. Available from: http://dphe.gov.bd/site/page/15fa0d7b-11f1-45c0-a684-10a543376873/Water-Quality-Parameters-. Cited 4 June 2021.

[147] [Kingdom of Bhutan]. National Environment Commission. Water quality standards 2018. Thimphu: National Environment Commission; 2018. Available from: http://extwprlegs1.fao.org/docs/pdf/bhu202080.pdf. Cited 5 June 2021.

[148] [State of Brunei]. Ministry of Development. Public Works Department. Water. FAQs. 2015. Available from: http://ebis.pwd.gov.bn/jkr_water/web/view/?id=ART00014-2007. Cited 24 July 2018.

[149] Kingdom of Cambodia. Ministry of Industry Mines and Energy. Drinking water quality standards. Phnom Penh: Ministry of Industry Mines and Energy; January 2004. Available from: http://rdic.org/wp-content/uploads/2014/12/MIME-Drinking-Water-Quality-Standards-2004-en.pdf. Cited 6 June 2021.

[150] Standardization Administration of China. Standards for drinking water quality. National Standard of the People’s Republic of China. GB 5749–2006. Replace: GB 5749–1985. Beijing: Standardization Administration of China; 29 December 2006. Available from: https://www.iwa-network.org/filemanager-uploads/WQ_Compendium/Database/Selected_guidelines/016.pdf. Cited 19 October 2021.

[151] საქართველოს მთავრობის. სასმელი წყლის ტექნიკური რეგლამენტის დამტკიცების შესახებ. დადგენილება №58. Tbilisi; 15 January 2014. Available from: https://matsne.gov.ge/ka/document/view/2196792?publication=0. Cited 10 June 2021.

[152] Bureau of Indian Standards. Indian standard, drinking water—specification (Second revision). IS 10500: 2012. New Delhi: Bureau of Indian Standards; May 2012. Available from: http://cgwb.gov.in/documents/wq-standards.pdf. Cited 12 June 2021.

[153] [Republic of Indonesia]. Menteri Kesehatan. Peraturan Menteri Kesehatan Nomor 492 / Menkes / Per / IV / 2010 Tanggal 19 April 2010 tentang Persyaratan Kualitas Air Minum. Jakarta: Menteri Kesehatan; 19 April 2010. Available from: http://repository.usu.ac.id/bitstream/123456789/26088/1/Appendix.pdf. Cited 26 July 2018.

[154] Islamic Republic of Iran. Institute of Standards and Industrial Research of Iran. Drinking water—physical and chemical specifications. ISIRI 1053 5th. revision. Tehran: Institute of Standards and Industrial Research of Iran; 2010. Available from: www.environment-lab.ir/standards/water-drink-standard-1053.pdf. Cited 12 June 2021.

[155] Central Organization for Standardization and Quality Control. Iraqi standard of drinking water. IS 417. Second modification. Baghdad: Central Organization for Standardization and Quality Control; 2009. [Not obtained. May be available for purchase online at https://www.cosqc.gov.iq/].

[156] Mahdi BA, Moyel MA, Jaafar RS. Adopting the Water Quality Index to assess the validity of groundwater in Al-Zubair city southern Iraq for drinking and human consumption. Eco Env & Cons. 2021; 27: 73–79.

[157] [State of Israel]. תקנות בריאות העם) איכותם התברואית של מי־שתיה ומיתקני מי שתיה. Tel Aviv; 16 May 2016. Available from: https://www.health.gov.il/LegislationLibrary/Briut47.pdf. Cited 12 June 2021.

[158] [Japan]. [Ministry of Health, Labour and Welfare]. Drinking water quality standards in Japan. April 2015. Available from: https://www.mhlw.go.jp/english/policy/health/water_supply/dl/4a.pdf. Cited 13 June 2021.

[159] Jordan Standards and Metrology Organization. Water–drinking water. JS 286:2008. Fifth edition. Amman: Jordan Standards and Metrology Organization; 2008. Available from: http://gis.nacse.org/rewab/docs/JS286_Drinking_Water_2008_ar.pdf. Cited 13 June 2021.

[160] [Republic of Kazakhstan]. Министр национальной экономики Республики Казахстан. Об утверждении Санитарных правил "Санитарно-эпидемиологические требования к водоисточникам, местам водозабора для хозяйственно-питьевых целей, хозяйственно-питьевому водоснабжению и местам культурно-бытового водопользования и безопасности водных объектов". Astana; 22 April 2015; No. 10774. Available from: https://adilet.zan.kz/rus/docs/V1500010774. Cited 14 June 2021.

[161] Beatona. Kuwait Official Environmental Portal. Water quality index in Kuwait. 28 January 2011. Available from: http://www.beatona.net/en/knowledge-hub/article/water-quality-index. Cited 21 June 2021.

[162] [Kyrgyz Republic]. Министерство Здравоохранения Кыргызской Республики. Питьевая вода. Гигиенические требования к качеству воды. Централизованных систем питьевого водоснабжения. Контроль качества санитарно—эпидемиологические правила и нормативы санпин 2.1.4.002–03. Bishkek: Министерство Здравоохранения Кыргызской Республики; 19 March 2004. Available from: http://extwprlegs1.fao.org/docs/texts/kyr94906.doc. Cited 21 June 2021.

[163] Lao People’s Democratic Republic. Prime Minister’s Office. Water Resources and Environment Administration. Agreement on the national environmental standards. No2734 /PMO.WREA. Vientiane: Water Resources and Environment Administration; 7 December 2009. Available from: http://sdms.gov.la/kcfinder/upload/files/5.11 AGREEMENT—National Environmental Standards—7.12.2009.pdf. Cited 27 July 2018.

[164] Lebanese Standards Institution. Document number NL 161. 1999. Available from: http://extwprlegs1.fao.org/docs/pdf/leb17709.pdf. Cited 22 June 2021.

[165] Malaysia. Kementerian Kesihatan Malaysia. Engineering Services Division. National standard for drinking water quality. Dokumen D1. Revised December 2000, Second Version, January 2004. Kuala Lumpur: Kementerian Kesihatan Malaysia; 2004. Available from: http://extwprlegs1.fao.org/docs/pdf/mal189903.pdf. Cited: 31 May 2021.

[166] [Republic of Maldives]. Environmental Protection Agency. Supply water quality standard. Male’: Environmental Protection Agency; 2017. Available from: http://files.epa.gov.mv/file/1271. Cited 31 May 2021.

[167] Монгол Улсын Засгийн Газрын Тохируулагч Агентлаг. Ундны ус. Эрүүл ахуйн шаардлага, чанар, аюулгүй байдлын үнэлгээ. MNS 0900: 2018. Ulaan Bator: Монгол Улсын Засгийн Газрын Тохируулагч Агентлаг; 2018. [Not obtained. Available for purchase online from https://eStandard.gov.mn/].

[168] Монгол Улсын Засгийн Газрын Тохируулагч Агентлаг. Стандартын мэдээллийн нэгдсэн сан. Available from: https://estandard.gov.mn/. Cited 19 October 2021.

[169] Hofmann J, Watson V, Scharaw B. Groundwater quality under stress: contaminants in the Kharaa River basin (Mongolia). Environ Earth Sci. 2015;73: 629–648. doi: 10.1007/s12665-014-3148-2

[170] Myanmar. Occupational and Environmental Health Division. Drinking water quality standards. Naypyitaw: Occupational and Environmental Health Division; 2018. [Not obtained].

[171] Thi MK. Drinking water quality standards out this year. Myanmar Times; 20 July 2018. Available from: https://www.mmtimes.com/news/drinking-water-quality-standards-out-year.html. Cited 19 October 2021.

[172] Norwegian Institute for Water Research. Integrated water resources management in Myanmar. Water usage and introduction to water quality criteria for lakes and rivers in Myanmar. Preliminary report. Rapport L.Nr. 7163–2017. Oslo: Norwegian Institute for Water Research; 31 May 2017. Available from: https://www.niva.no/en/projectweb/myanmar/publications/_/attachment/download/093a59d4-0ee9-47ee-841f-5a510dddea6b%3A57f9381314ea6737f884cf69d58e95bdf1480c68/12377_report-generell_del-final-08062017.pdf. Cited 27 June 2021.

[173] Government of Nepal. Ministry of Physical Planning and Works. National drinking water quality standards 2005. Kathmandu: Ministry of Physical Planning and Works.; 2005. Available from: http://mowss.gov.np/assets/uploads/files/NDWQS_2005_Nepal.pdf. Cited 27 June 2021.

[174] Directorate General for Standards and Metrology (DGSM). Un Bottled Drinking Water. OS 8/2012. Muscat: Directorate General for Standards and Metrology; 2012. Available from: https://www.pdo.co.om/hseforcontractors/LegalRequirements/OS 8-2012-E-Unbottled Drinking Water Standard.pdf. Cited 30 June 2021.

[175] Government of Pakistan. Ministry of the Environment. National standards for drinking water quality. The Gazette of Pakistan; 26 November 2010. Available from: http://environment.gov.pk/images/rules/SRO2010NEQSAirWaterNoise.pdf. Cited 1 July 2021.

[176] Republic of the Philippines. Department of Health. Philippine national standards for drinking water of 2017. Manila: Department of Health; 23 June 2017. Available from: https://www.mcwd.gov.ph/wp-content/uploads/2017/07/addendum-bulk-water-annexB.pdf. Cited 31 July 2018.

[177] KAHRAMAA (The Qatar General Water and Electricity Corporation). Overview on: KAHRAMAA drinking water quality requirements. Doha: KAHRAMAA; 2014. Available from: https://www.km.qa/MediaCenter/Publications/KAHRAMAA%20Drinking%20Water%20Quality%20Requirment.pdf. Cited 4 July 2021.

[178] Kingdom of Saudi Arabia. Royal Commission Environmental Regulations 2015, Environmental Control Department. Drinking water quality standards. RCER -2015 Volume 1. Riyadh: Environmental Control Department; 2015. Available from: https://www.mwa.co.th/download/prd01/iDW_standard/Saudi_Arabia_Water_Standard_2015.pdf. Cited 9 July 2021.

[179] Singapore statutes online. The Schedule. Regulations 2, 3, 5, 8 and 9. Drinking water quality standards. Singapore; 14 February 2019. Available from: https://www.mwa.co.th/download/prd01/iDW_standard/Singapore_Drinking_Water_Standard.pdf. Cited 12 July 2021.

[180] [Republic of Korea (South Korea)]. Meogneunmul Sujilgijun. Sigsuyong. Seoul; 2018. Available from: http://easylaw.go.kr/CSP/CnpClsMain.laf?popMenu=ov&csmSeq=536&ccfNo=2&cciNo=1&cnpClsNo=1. Cited 22 April 2018.

[181] Busan Metropolitan City Office of the Water Supply. Water quality standards for drinking water. Available from: https://www.busan.go.kr/water_en/Flammableorganic. Cited 14 July 2021.

[182] [Democratic Socialist Republic of Sri Lanka]. National environmental (ambient water quality) regulations, No. 01 of 2019. National Environmental Act, no. 47 of 1980. The Gazette of the Democratic Socialist Republic of Sr Lanka; 4 November 2019; No. 2148/20. Available from: http://extwprlegs1.fao.org/docs/pdf/srl202534.pdf. Cited 23 July 2021.

[183] The Syrian Arab Organization for Standardization & Metrology. Drinking water—(second revision). SASMO 45. Damascus: The Syrian Arab Organization for Standardization & Metrology; 7 May 2007. [Not obtained. Available for purchase online from http://www.sasmo.org.sy/.]

[184] The Syrian Arab Organization for Standardization & Metrology. Syrian Standards Guide. Available from: http://www.sasmo.org.sy/standard/guide. Cited 20 October 2021.

[185] Yachiyo Engineering Co., Ltd. The study on solid waste management at local cities in the Syrian Arab Republic. Final report—supporting report. Appendix 5—environmental baseline survey (Lattakia). Tokyo: Yachiyo Engineering Co., Ltd; 2002. Available from: https://openjicareport.jica.go.jp/pdf/11688769_06.pdf. Cited 24 July 2021.

[186] [Republic of China (Taiwan)]. Drinking Water Management Act. Drinking Water Quality Standards. Taipei; 10 January 2017. Available from: https://law.moj.gov.tw/ENG/LawClass/LawAll.aspx?pcode=O0040019. Cited 31 July 2021.

[187] Aliev S, Shodmonov P, Babakhanova N, Schmoll O. Rapid assessment of drinking-water quality in the Republic of Tajikistan. Geneva: World Health Organization; 2010. Available from: https://washdata.org/file/488/download. Cited 17 April 2018.

[188] Kingdom of Thailand. Notification of the Ministry of Natural Resources and Environment, B.E. 2551 (2008) issued under the Groundwater Act, B.E. 2520 (1977). Royal Government Gazette. 21 May 2008; Vol. 125, Special Part 85D. [Not obtained].

[189] Electricity Generating Authority of Thailand. มาตรฐานคุณภาพสิ่งแวดล้อม. Bangkok: Electricity Generating Authority of Thailand; 2015. Available from: https://maemohmine.egat.co.th/standard_env.pdf. Cited 4 August 2018.

[190] Michael H. Drinking-water quality assessment and treatment in East Timor. Case study: Tangkae. Thesis. Perth: The University of Western Australia; 2006. Available from: http://www.education.uwa.edu.au/__data/assets/pdf_file/0004/1637464/Michael_2007.pdf. Cited 31 July 2021.

[191] [Republic of Turkey]. Içme suyu temin edilen sularin kalitesi ve aritilmasi hakkinda yönetmelik. Resmî gazete. 6 July 2019; sayı: 30823. Available from: https://www.resmigazete.gov.tr/eskiler/2019/07/20190706-8.htm. Cited 7 August 2021.

[192] Republic of Turkey. Içme suyu temin edilen sularin kalitesi ve aritilmasi hakkinda yönetmelik. Ek-1. Kategorı göre su kalıte standartlari. Resmî gazete. 6 July 2019; sayı: 30823. Available from: https://www.resmigazete.gov.tr/eskiler/2019/07/20190706-8-1.pdf. Cited 7 August 2021.

[193] Türkmenstandartlary. Agyz suwlary gazlanan, gazlanmadyk, ýakymly ysly, ýakymly yssyz. Umumy tehniki şertler TDS 576–2001 deregine. Ashgabat: Türkmenstandartlary; 2016. [Not obtained. May be available for purchase in person in Ashgabat.]

[194] Türkmenstandartlary. Türkmenistanyň kadalaşdyryjy resminamalaryň. Available from: https://turkmenstandartlary.gov.tm/index.php?page=tm_ukazateli_1. Cited 20 October 2021.

[195] Government of Turkmenistan. Закон Tуркменистана о питьевой воде. Ashgabat; 25 September 2010; №136-IV. Available from: http://faolex.fao.org/docs/texts/tuk105947.doc. Cited 7 August 2021.

[196] Government of Abu Dhabi. The water quality regulations. 4th ed. 2014 January. (WA-705). Available from: https://jawdah.qcc.abudhabi.ae/en/Registration/QCCServices/Services/STD/ISGL/ISGL-LIST/WA-705.pdf. Cited 24 August 2021.

[197] Утверждаю. Гигиенические критерии и контроль качества воды централизованных систем хозяйственно-питьевого водоснабжения населения узбекистана. Ниязматов Б.И. 01.06.2006 г., № 0211–06. Tashkent: Утверждаю; 2006. Available from: http://lex.uz/pages/getpage.aspx?lact_id=1934624. Cited 19 April 2018.

[198] Socialist Republic of Vietnam. Ministry of Health. Department of Preventive Medicine & Environment. National technical regulation on drinking water quality. QCVN 01: 2009/BYT. Hanoi: Ministry of Health; 17 June 2009. Available from: https://www.mwa.co.th/download/prd01/iDW_standard/Vietnam_Drinking_Water_Quality_2009.pdf. Cited 12 July 2021.

[199] [Republic of Yemen]. [Office of the Council of Ministers] [Public Office of Water Resources] [Specific standards of water for Yemen. Public drinking water]. Sana’a; 1999. Available from: https://yemen-nic.info/files/water/studies/Public_drinking_water.pdf. Cited 21 October 2021.

[200] Australian Government. National Health and Medical Research Council. Australian drinking water guidelines 6 2011, version 3.4 updated October 2017. Canberra: National Health and Medical Research Council; October 2017. Available from: https://www.nhmrc.gov.au/sites/default/files/documents/reports/aust-drinking-water-guidelines.pdf. Cited 4 June 2021.

[201] Federated States of Micronesia. Consolidated legislation—revised code 1999. Trust Territory environmental quality protection act general provisions. § 103 Definitions, (4), (6). Palikir; 1999. Available from: http://www.paclii.org/cgi-bin/disp.pl/fm/legis/consol_act_1999/tteqpagp826/tteqpagp826.html?stem=0&synonyms=0&query=drinking%20water. Cited 10 September 2021.

[202] [Republic of Fiji]. Ministry of Health. Fiji national drinking water standards. Suva: Ministry of Health; 2011. [Not obtained].

[203] [Republic of Fiji]. Ministry of Education, National Heritage, Culture & Arts. Minimum standards on water, sanitation and hygiene (WASH) in schools infrastructure. Suva: Ministry of Education, National Heritage, Culture & Arts; September 2012. Available from: https://livelearn.org/assets/media/docs/resources/Fiji_MEHA_WinS_Standards.pdf. Cited 10 June 2021.

[204] [Republic of Fiji]. Trade standards and quality control decree 1992 (Decree no. 24 of 1992). Trade standard (Bottled water standard) order 2004. Legal notice no. 72. Extraordinary Fiji Islands Government Gazette Supplement; 2004: No. 29: 235–251. Available from: http://www.mitt.gov.fj/images/Trade_Standards_Bottle_Water_Standards_Order_2004.pdf. Cited 3 March 2018.

[205] Republic of the Marshall Islands. Environmental Protection Authority. Public water supply regulations 1994. Majuro: Environmental Protection Authority; 1994. Available from: http://rmicourts.org/wp-content/uploads/Public-Water-Supply-Regulations-1994.pdf. Cited 23 June 2021.

[206] Republic of Nauru. National water, sanitation and hygiene policy. Updated September 2012. Yaren; 2012: Available from: http://extwprlegs1.fao.org/docs/pdf/nau181071.pdf. Cited 27 June 2021.

[207] [New Zealand]. Ministry of Health. Drinking-water Standards for New Zealand 2005. (Revised 2018). Wellington: Ministry of Health; December 2018. Available from: https://www.mwa.co.th/download/prd01/iDW_standard/New_Zealand_Drinking_Water_Standard_2018.pdf. Cited 29 June 2021.

[208] [Republic of Palau.] Chapter 2401–51. Public water supply system regulations. Koror City; 26 May 1996. Available from: http://extwprlegs1.fao.org/docs/pdf/pau32797.pdf. Cited 1 July 2021.

[209] Independent State of Papua New Guinea. Public health (drinking water) regulation 1984. Public Health Act 1973. This reprint of this Statutory Instrument incorporates all amendments, if any, made before 25 November 2006 and in force at 1 July 2001. Port Moresby; 25 November 2006. Available from: http://faolex.fao.org/docs/texts/png53729.doc. Cited 2 July 2021.

[210] Samoa Water Authority. Samoa national drinking water standards. Apia: Samoa Water Authority; 2016. [Not obtained].

[211] Samoa Water Authority. Corporate plan 2021–2024. Apia: Samoa Water Authority; 2021. Available from: https://www.palemene.ws/wp-content/uploads/SWA-Corporate-Plan-2021-2024-03072020.pdf. Cited 10 September 2021.

[212] Imo T, Amosa P, Vaurasi V, Latu F. Chemical contamination in a typical independent water scheme (IWS) catchment. Int J Environ Sci Develop. 2019; 10:445–449. doi: 10.18178/ijesd.2019.10.12.1214

[213] [Solomon Islands.] Pure food (food control) regulations 2010. Legal notice no. 154. Solomon Islands Gazette. 25 November 2010; Supplement S.I. No 70. Available from: http://www.paclii.org/sb/legis/sub_leg/pfa1996pfcr2010461.pdf. Cited 31 August 2021.

[214] White I, Falkland T, Fatai T. Vulnerability of groundwater in Tongatapu, Kingdom of Tonga: Groundwater monitoring and monitoring assessment. Canberra: Australian National University; June 2009. Available from: https://tonga-data.sprep.org/system/files/Tongatapu%20Groundwater%20Vulnerability%20%26%20Monitoring%20Report%2C%2022Sep09.pdf. Cited 30 July 2021.

[215] [Tuvalu]. Report of the Auditor-General. Performance audit report on access to safe drinking water. Funafuti; 24 October 2011. Available from: http://www.tuvaluaudit.tv/wp-content/uploads/2014/05/Final-Safe-Drinking-Water-Report.docx. Cited 7 August 2021.

[216] Republic of Vanuatu. Water supply act [CAP 24]. National drinking water quality standards, order no. 51 of 2019. Port Vila; 13 May 2019. Available from: https://mol.gov.vu/images/News-Photo/water/DoWR_File/Monitoring_Evaluation/Official_Gazette_No_26_of_2019_dated_13_June_2019_1.pdf. Cited 31 August 2021.

[217] [Republic of Albania]. Vendim nr. 379, datë 25.5.2016 Për miratimin e rregullores “cilësia e ujit të pijshëm”. Tirana; 25 May 2016. Available from: http://extwprlegs1.fao.org/docs/pdf/alb163693.pdf. Cited 4 June 2021.

[218] [Principality of Andorra]. Decret pel qual s’aprova el reglament relatiu als criteris sanitaris de la qualitat de l’aigua destinada al consum humà, del 17 d’octubre del 2007. Butlletí Oficial del Principat d’Andorra. 24 October 2007: 88:4649–4690. Available from: https://www.bopa.ad/bopa/019088/Documents/4F132.pdf. Cited 5 June 2021.

[219] [Republic of Austria]. 304. Verordnung des Bundesministers für soziale Sicherheit und Generationen über die qualität von wasser für den menschlichen gebrauch (trinkwasserverordnung–TWV). CELEX-Nr.: 398L0083. Bundesgesetzblatt für die Republik Österreich. 21 August 2001; 1805–1822. Available from: https://www.ris.bka.gv.at/Dokumente/BgblPdf/2001_304_2/2001_304_2.pdf. Cited 4 June 2021.

[220] [Republic of Belarus]. Министерствo Здравоохранения. Постановление Министерства здравоохранения Республики Беларусь от 15.12.2015 N 123 "Об утверждении Санитарных норм и правил "Требования к питьевой воде, расфасованной в емкости", Гигиенического норматива "Требования к безопасности питьевой воды, расфасованной в емкости" и признании утратившим силу постановления Министерства здравоохранения Республики Беларусь от 29 июня 2007 г. N 59". Minsk: Ministry of Health; 15 December 2015. Available from: http://extwprlegs1.fao.org/docs/pdf/blr159385.pdf. Cited 5 June 2021.

[221] [Kingdom of Belgium]. Ministere de la Communaute Flamande. 13 Decembre 2002.—Arrêté du gouvernement flamand portant réglementation relative à la qualité et la fourniture des eaux destinées à la consommation humaine. Moniteur Belge. 28 January 2003; 2923–2938. Available from: https://www.cstc.be/index.cfm?dtype=services&doc=Arrete_13_12_2002.pdf. Cited 5 June 2021.

[222] [Bosnia and Herzegovina]. Pravilnik o zdravstvenoj ispravnost vode za piće. Službeni glasnik BiH. 21 January 2010; broj: 40/10, 43/10, 30/12. Available from: http://extwprlegs1.fao.org/docs/pdf/bih148927.pdf. Cited 4 June 2021.

[223] [Republic of Bulgaria]. Наредба No 9 oт 16.03.2001 г. за качествого на водата, предназначена за питейно-битови цели. Sofia; 16 March 2001. Available from: http://extwprlegs1.fao.org/docs/pdf/bul33621.pdf. Cited 4 June 2021.

[224] [Croatian Republic]. Uredbu o standard kakvoće voda. Vlada Republike Hrvatske. 9 October 2019; NN 96/2019. Available from: https://narodne-novine.nn.hr/clanci/sluzbeni/2019_10_96_1879.html. Cited 7 June 2021.

[225] [Republic of Cyprus]. Αριθμός 87(Ι) του 2001. Νομοσ που προβλεπει για την παρακολουθηση και τον ελεγχο τησ ποιοτητασ του νερου ανθρωπινησ καταναλωσησ. E.E. ParI(I). 4 May 2001; Ar. 3496. Available from: http://www.cylaw.org/nomoi/arith/2001_1_087.pdf. Cited 7 June 2021.

[226] [Czech Republic]. Vyhláška, kterou se mění vyhláška č. 252/2004 sb., kterou se stanoví hygienické požadavky na pitnou a teplou vodu a četnost a rozsah kontroly pitné vody. Sbírka zákonů ČR. 29 May 2014; 83/2014. Available from: http://extwprlegs1.fao.org/docs/pdf/cze141277.pdf. Cited 8 June 2021.

[227] [Kingdom of Denmark]. Miljø- og Fødevareministeriet. Bekendtgørelse om vandkvalitet og tilsyn med vandforsyningsanlæg. BEK nr 1070 af 28/10/2019. Miljø og Fødevaremin 2019; 2019–14154. Available from: https://www.retsinformation.dk/eli/lta/2019/1070. Cited 8 June 2021.

[228] [Republic of Estonia]. Minister of Social Affairs. Quality and control requirements and analysis methods for drinking water. Regulation no. 82 of the Minister of Social Affairs of 31 July 2001 (RTL 2001, 100, 1369), entered into force 1 June 2002. Tallinn: Minister of Social Affairs; 1 June 2002. Available from: http://extwprlegs1.fao.org/docs/texts/est98585E.doc. Cited 9 June 2021.

[229] [Republic of Finland]. Social- och hälsovårdsministeriet. Förordning om ändring av social- och hälsovårdsministeriets förordning om kvalitetskrav på och kontrollundersökning av hushållsvatten. Helsinki: Social- och hälsovårdsministeriet; 2 June 2014. Available from: http://extwprlegs1.fao.org/docs/pdf/fin180866.pdf. Cited 10 June 2021.

[230] République Française. Arrêté du 4 août 2017 modifiant plusieurs arrêtés relatifs aux eaux destinées à la consommation humaine pris en application des articles R. 1321–2, R. 1321–3, R. 1321–10, R. 1321–15, R. 1321–16, R. 1321–24, R. 1321–84, R. 1321–91 du code de la santé publique. JORF. 17 August, 2017; No 0191:Texte No. 29. Available from: https://www.legifrance.gouv.fr/jorf/id/JORFTEXT000035427495. Cited 22 October 2021.

[231] INERIS. Synthèse des valeurs réglementaires pour les substances chimiques, en vigueur dans l’eau, les denrées alimentaires et dans l’air en France au 31 décembre 2017. Rapport INERIS-DRC-17-164559-10404A. Paris: INERIS; 3 March 2018. Available from: https://www.actu-environnement.com/media/pdf/news-30940-rapport-ineris-valeurs.pdf. Cited 10 June 2021.

[232] [Federal Republic of Germany]. Erste verordnung zur änderung der grundwasserverordnung. Berlin; 2 March 2017. Available from: https://www.bmu.de/fileadmin/Daten_BMU/Download_PDF/Gesetze/grundwasser_vo.pdf. Cited 10 June 2021.

[233] [Greek Republic]. Αποφασεισ. Αριθμ. Γ1(δ)/ ΓΠ οικ.67322. Ποιότητα νερού ανθρώπινης κατανάλωσης σε συμμόρφωση προς τις διατάξεις της Οδηγί-ας 98/83/ΕΚ του Συμβουλίου της Ευρωπαϊκής Ένωσης, της 3ης Νοεμβρίου 1998 όπως τροπο-ποιήθηκε με την Οδηγία (ΕΕ) 2015/1787 (L260, 7.10.2015). Athens: 19 September, 2017; 2:3282. Available from: http://www.moh.gov.gr/articles/health/dieythynsh-dhmosias-ygieinhs/ygieinh-periballontos/prostasia-poiothtas-ydatwn/prostasia-neroy-anthrwpinhs-katanalwshs/4966-ekdosh-k-y-a-gia-thn-poiothta-toy-neroy-anthrwpinhs-katanalwshs-2017?fdl=12221. Cited 11 June 2021.

[234] [Republic of Hungary]. Az ivóvízkivételre használt vagy ivóvízbázisnak kijelölt felszíni víz, valamint a halak életfeltételeinek biztosítására kijelölt felszíni vizek szennyezettségi határértékeirõl és azok ellenõrzésérõl. 6/2002. (XI. 5.) KvVM rendelet. Budapest: June 2002. Available from: http://extwprlegs1.fao.org/docs/texts/hun35417.doc. Cited 11 June 2021.

[235] [Republic of Iceland]. Reglugerð um neysluvatn. 536/2001. Reyjavik; 28 June 2002. Available from: https://www.reglugerd.is/reglugerdir/allar/nr/536-2001. Cited 11 June 2021.

[236] [Ireland]. European Union (drinking water) regulations 2014. Statutory instruments. S.I. no. 122 of 2014. Dublin; 27 February 2014. Available from: http://extwprlegs1.fao.org/docs/pdf/ire134269.pdf. Cited 12 June 2021.

[237] [Italian Republic]. Attuazione della direttiva 98/83/CE relativa alla qualità delle acque destinate al consumo umano. Gazzetta Ufficiale. 3 March 2001; Supplement n. 52. Available from: https://www.physico.eu/pdf/direttiva-europea-98-83-ce.pdf. Cited 12 June 2021.

[238] [Italian Republic]. Ministero Della Salute. Decreto 14 novembre 2016. Modifiche all’allegato I del decreto legislativo 2 febbraio 2001, n. 31, recante: «Attuazione della direttiva 98/83/CE relativa alla qualita’ delle acque destinate al consumo umano». Rome: Ministero Della Salute; 14 November 2016. Available from: http://extwprlegs1.fao.org/docs/pdf/ita162901.pdf. Cited 12 June 2021.

[239] Republikës ë Kosovës. Udhëzimi administrativ nr. 16/2012 për cilësinë e ujit për konsum nga njeriu. Pristina; 14 December 2012. Available from: http://www.kryeministri-ks.net/repository/docs/Udhezim_Administrativ__Nr.16_.pdf. Cited 14 June 2021.

[240] [Republic of Latvia]. Dzeramā ūdens obligātās nekaitīguma un kvalitātes prasības, monitoringa un kontroles kārtība. Ministru kabineta noteikumi Nr. 671. Latvijas Vēstensis. 16 November 2017; 228(605): prot. Nr. 57 35. §. Available from: https://likumi.lv/ta/id/295109-dzerama-udens-obligatas-nekaitiguma-un-kvalitates-prasibas-monitoringa-un-kontroles-kartiba. Cited 22 June 2021.

[241] [Principality of Liechtenstein]. Verordnung vom 28. August 2018, über die abänderung der trinkwasserverordnung. Liechtensteinisches Landesgesetzblatt. 4 September 2018; nr. 177. Available from: https://www.gesetze.li/chrono/2018177000. Cited 22 June 2021.

[242] Lietuvos Respublikos sveikatos apsaugos ministro. Geriamojo vandens saugos ir kokybės reikalavimai. Lietuvos higienos norma hn 24:2017. Vilnius; 27 October 2017. Available from: https://www.esavadai.lt/dokumentai/6164-hn-242017-geriamojo-vandens-saugos-ir-kokybes-reikalavimai-pakeitimai-nuo-2020-05-14/. Cited 22 June 2021.

[243] [Grand Duchy of Luxembourg]. Règlement grand-ducal du 7 juillet 2017 modifiant le règlement grand-ducal modifié du 7 octobre 2002 relatif à la qualité des eaux destinées à la consommation humaine. Journal Officiel du Grand-Duché de Luxembourg. 12 July 2017; mémorial a n° 637. Available from: http://data.legilux.public.lu/file/eli-etat-leg-rgd-2017-07-07-a637-jo-fr-pdf.pdf. Cited 22 June 2021.

[244] [Republic of Malta]. Regolamenti tal-2017 li jemendaw ir-regolamenti dwar ilma maħsub għall-konsum mill-bniedem. Tal-Gazzetta tal-Gvern ta’ Malta. 24 October 2017; Supplement. Taqsima B: 2117–2140. Available from: http://extwprlegs1.fao.org/docs/pdf/mlt84621.pdf. Cited 23 June 2021.

[245] Республика Молдова. Закон № 182 от 19-12-2019 о качестве питьевой воды. Monitorul Oficial. 3 January 2020; № 1–2 статья № 2. Available from: http://extwprlegs1.fao.org/docs/pdf/mol196614.pdf. Cited 24 June 2021.

[246] [Principality of Monaco]. Ordonnance souveraine n° 6.696 du 7 Décembre 2017 relative à la qualité et à la surveillance de l’eau potable de consommation humaine distribuée. Journal de Monaco. 15 December 2017; 8360. Available from: https://journaldemonaco.gouv.mc/Journaux/2017/Journal-8360/Ordonnance-Souveraine-n-6.696-du-7-decembre-2017-relative-a-la-qualite-et-a-la-surveillance-de-l-eau-potable-de-consommation-humaine-distribuee. Cited 24 June 2021.

[247] [Republic of Montenegro]. Pravilnik o bližim zahtjevima koje u pogledu bezbjednosti treba da ispunjava voda za piće. Podgorica; 25 April 2015. Available from: http://extwprlegs1.fao.org/docs/pdf/mne139310.pdf. Cited 26 June 2021.

[248] [Kingdom of the Netherlands]. Besluit van 23 mei 2011, houdende bepalingen inzake de productie en distributie van drinkwater en de organisatie van de openbare drinkwatervoorziening (Drinkwaterbesluit). Staatsblad van het Koninkrijk der Nederlanden. 21 June 2011; 293. Available from: https://zoek.officielebekendmakingen.nl/stb-2011-293.html. Cited 14 July 2021.

[249] [Kingdom of Norway]. Helse- og omsorgdepartementet. Forskrift om vannforsyning og drikkevann (drikkevannsforskriften). I 2016 hefte 19 s 3142. Oslo; 22 December 2016. Available from: https://lovdata.no/dokument/LTI/forskrift/2016-12-22-1868. Cited 29 June 2021.

[250] [Republic of Poland]. Ministra Zdrowia. Rozporządzenie Ministra Zdrowia z dnia 7 grudnia 2017 r. w sprawie jakości wody przeznaczonej do spożycia przez ludzi. Dziennik Ustaw Rzeczypospolitej Polskiej. 11 December 2019; Poz. 2294. Available from: http://extwprlegs1.fao.org/docs/pdf/pol182571.pdf. Cited 3 July 2021.

[251] [Portuguese Republic]. Ambiente decreto-lei n.° 152/2017 de 7 de dezembro. Diário da República, 1.ª série. 7 December 2017; N.° 235:6555–6576. Available from: http://extwprlegs1.fao.org/docs/pdf/por176166.pdf. Cited 4 July 2021.

[252] [Republic of North Macedonia]. Правилник за барања за безбедност и квалитет на водата за пиење. Службен весник на РМ. 2 October 2018; бр. 183. Available from: http://extwprlegs1.fao.org/docs/pdf/mac187611.pdf. Cited 5 July 2021.

[253] [Romania]. Lege nr. 458 din 8 iulie 2002 privind calitatea apei potabile—republicare la data 31-Jan-2019. Bucharest; 31 January 2019. Available from: https://www.cupfocsani.ro/public/upload/documents/legislatie/pdf-yvnq6gfo.pdf. Cited 5 July 2021.

[254] [Russian Federation]. Постановление Главного государственного санитарного врача Российской Федерации от 26 сентября 2001 г. N 24. Moscow; 31 October 2001. Available from: http://extwprlegs1.fao.org/docs/pdf/rus187270.pdf. Cited 5 July 2021.

[255] Repubblica di San Marino. Codice ambientale. Decreto delegato 27 aprile 2012 n. 44. San Marino; 27 April 2012. Available from: https://www.consigliograndeegenerale.sm/on-line/home/in-evidenza-in-home-page/documento17050172.html. Cited 9 July 2021.

[256] [Republic of Serbia]. Pravilnik o higijenskoj ispravnosti vode za piće. Službeni glasnik. 25 April 2019; SRJ 42/98, 44/99, 28/2019. Available from: https://www.tehnologijahrane.com/pravilnik/pravilnik-o-higijenskoj-ispravnosti-vode-i. Cited 14 July 2021.

[257] [Slovak Republic]. Nariadenie vlády Slovenskej republiky, ktorým sa ustanovujú požiadavky na vodu určenú na ľudskú spotrebu a kontrolu. Bratislava; 10 May 2006. Available from: http://www.zakonypreludi.sk/zz/2006-354#f6480329. Cited 14 July 2021.

[258] [Republic of Slovenia]. Zakonodaja pravilnik o pitni vodi (UL RS št. št. 19/04, št. 35/04, št. 26/06, št. 92/06, št. 25/09) pravilnik. Priloga I: Parametri in mejne vrednosti parametrov. Ljubljana; 2015. Available from: http://www.pisrs.si/Pis.web/npb/2017-01-2386-2004-01-0865-npb6-p1.pdf. Cited 15 July 2021.

[259] [Kingdom of Spain]. Real Decreto 140/2003, de 7 de febrero, por el que se establecen los criterios sanitarios de la calidad del agua de consumo humano. Boletín Oficial del Estado. 21 February 2003; 45. Available from: https://www.boe.es/buscar/act.php?id=BOE-A-2003-3596. Cited 23 July 2021.

[260] [Kingdom of Sweden]. Livsmedelsverket. Livsmedelsverkets föreskrifter om ändring i Livsmedelsverkets föreskrifter (SLVFS 2001:30) om dricksvatten; beslutade den 21 september 2017. LIVSFS. 5 October 2017; H90. Available from: https://www.livsmedelsverket.se/globalassets/om-oss/lagstiftning/dricksvatten—naturl-mineralv—kallv/livsfs-2017-2_web.pdf. Cited 24 July 2021.

[261] Schweizerische Eidgenossenschaft. Eidgenossische Departement des Innern. Verordnung des EDI über Trinkwasser sowie Wasser in öffentlich zugänglichen Bädern und Duschanlagen. 817.022.11. Bern; 1 July 2021. Available from: https://www.fedlex.admin.ch/eli/cc/2017/153/de?print=true. Cited 24 July 2021.

[262] Міністерство Охорони Здоров’я України. Про затвердження Державних санітарних норм та правил “Гігієнічні вимоги до води питної, призначеної для споживання людиною”. Kiev: Ministerstvo okhoroni zdorov’ia ukraïni; 1 July 2010. Available from: https://zakon.rada.gov.ua/laws/show/z0452-10/print. Cited 24 August 2021.

[263] [United Kingdom of Great Britain and Northern Ireland]. Water, England. The private water supplies (England) regulations 2016. Statutory Instruments 2016 No. 618. London; 27 June 2016. Available from: http://www.legislation.gov.uk/uksi/2016/618/pdfs/uksi_20160618_en.pdf. Cited 24 August 2021.

[264] Kahle D, Wickham H. Spatial Visualization with ggplot2. The R Journal. 2013; 5(1): 144–161. Available from: http://journal.r-project.org/archive/2013-1/kahle-wickham.pdf. Cited 29 September 2021.

[265] World Health Organization. International standards for drinking-water. Geneva: World Health Organization; 1958. Available from: https://apps.who.int/iris/bitstream/handle/10665/43845/a91160.pdf?sequence=1&isAllowed=y. Cited 30 October, 2021.

[266] Kolthoff IM, Lingane JJ. Polarography. 2nd ed. 2 vol. New York: Interscience Publishers; 1952, pp. 542–545.

[267] Frisbie SH, Mitchell EJ, Yusuf AZ, Siddiq MY, Sanchez RE, Ortega R, et al. The development and use of an innovative laboratory method for measuring arsenic in drinking water from western Bangladesh. Environ Health Perspect. 2005. 113(9):1196–1204. doi: 10.1289/ehp.7974 16140627 PMC1280401

[268] World Health Organization. Guidelines for drinking-water quality. Geneva: World Health Organization; 1984. Available from: https://apps.who.int/iris/bitstream/handle/10665/252072/9241541687-eng.pdf?sequence=1&isAllowed=y. Cited 30 October, 2021.

[269] World Health Organization. Developing drinking-water quality regulations. Geneva: World Health Organization; 2018. Available from: https://apps.who.int/iris/bitstream/handle/10665/272969/9789241513944-eng.pdf?sequence=1&isAllowed=y. Cited 30 October, 2021.

[270] World Health Organization. Guidelines for drinking-water quality. Addendum to volume 1: Recommendations. 2nd ed. Geneva: World Health Organization; 1998. Available from: https://apps.who.int/iris/bitstream/handle/10665/63844/WHO_EOS_98.1.pdf?sequence=1&isAllowed=y. Cited 30 October, 2021.

[271] World Health Organization. Guidelines for drinking-water quality. First addendum to the third edition. Volume 1: Recommendations. 3rd ed. Geneva: World Health Organization; 2006. Available from: https://apps.who.int/iris/bitstream/handle/10665/43242/9241546743_eng.pdf?sequence=1&isAllowed=y. Cited 30 October, 2021.

[272] World Health Organization. Guidelines for drinking-water quality. 4th edition. Geneva: World Health Organization; 2011. Available from: https://apps.who.int/iris/bitstream/handle/10665/44584/9789241548151_eng.pdf?sequence=1&isAllowed=y. Cited 30 October, 2021.

[273] United States Environmental Protection Agency. 40 CFR Parts 141 and 142. National primary drinking water regulations; arsenic and clarifications to compliance and new source contaminants monitoring. Federal Register. 22 June 2000; 65(121). Available from https://www.govinfo.gov/content/pkg/FR-2000-06-22/html/00-13546.htm. Cited 8 April 2021.

[274] United States Environmental Protection Agency. Analytical methods support document for arsenic in drinking water. Washington, DC: United States Environmental Protection Agency; 1999. Available from: https://nepis.epa.gov/Exe/ZyNET.exe/2000JT4N.TXT?ZyActionD=ZyDocument&Client=EPA&Index=2000+Thru+2005&Docs=&Query=&Time=&EndTime=&SearchMethod=1&TocRestrict=n&Toc=&TocEntry=&QField=&QFieldYear=&QFieldMonth=&QFieldDay=&IntQFieldOp=0&ExtQFieldOp=0&XmlQuery=&File=D%3A%5Czyfiles%5CIndex%20Data%5C00thru05%5CTxt%5C00000010%5C2000JT4N.txt&User=ANONYMOUS&Password=anonymous&SortMethod=h%7C-&MaximumDocuments=1&FuzzyDegree=0&ImageQuality=r75g8/r75g8/x150y150g16/i425&Display=hpfr&DefSeekPage=x&SearchBack=ZyActionL&Back=ZyActionS&BackDesc=Results%20page&MaximumPages=1&ZyEntry=1&SeekPage=x&ZyPURL. Cited 27 October, 2021.

[275] United States Environmental Protection Agency. Drinking water standard for arsenic. EPA 815-F-00-015. Washington, DC: United States Environmental Protection Agency. January 2001. Available from: https://nepis.epa.gov/Exe/ZyPdf.cgi?Dockey=20001XXC.txt. Cited 27 October, 2021.

[276] United States Environmental Protection Agency. US EPA Science Advisory Board. Arsenic Rule Benefits Review Panel. Arsenic rule benefits analysis: an SAB review. EPA-SAB-EC-01-008. Washington, DC: United States Environmental Protection Agency; August 2001. Available from: https://nepis.epa.gov/Exe/ZyPDF.cgi?Dockey=P1004JZG.txt. Cited 2 April, 2021.

[277] California Environmental Protection Agency. Responses to major comments on technical support document public health goal for arsenic in drinking water. Sacramento: California Environmental Protection Agency. Available from: https://oehha.ca.gov/media/downloads/water/public-health-goal/ascom42304.pdf. Cited 6 August, 2017.

[278] American Public Health Association, American Water Works Association, Water Pollution Control Federation. Standard methods for the examination of water and wastewater. 17th ed. Washington, DC: American Public Health Association; 1989, pp. 3–74–3–76.

[279] United States. Title 40 of the Code of Federal Regulations, 1986. Guidelines establishing test procedures for the analysis of pollutants. 40 CFR Part 136 Appendix B. Available from: http://www.ecfr.gov/cgi-bin/text-idx?SID=1bf218b600f0e799ed4de0939b8fa837&mc=true&node=pt40.25.136&rgn=div5#ap40.25.136_17.b. Cited 8 July 2016.

[280] Frisbie SH, Mitchell EJ, Abualrub MS, Abosalem Y. Calculating the lowest reportable concentrations of toxic chemicals in the environment. Int J Appl Math Theor Phys. 2015; 1(1): 9–13.

[281] Itaya K, Ui M. A new micromethod for the colorimetric determination of inorganic phosphate. Clin Chim Acta. 1966; 14(3): 361–366. doi: 10.1016/0009-8981(66)90114-8 5970965

[282] Matsubara C, Yamamoto Y, Takamura K. Rapid determination of trace amounts of phosphate and arsenate in water by spectrophotometric detection of their heteropoly acid-Malachite Green aggregates following preconcentration by membrane filtration. Analyst. 1987; 112: 1257–1260. doi: 10.1039/an9871201257 3425928

[283] Wu QF, Liu PF. A highly sensitive spectrophotometric method for determination of micro amounts of arsenic. Talanta. 1983; 30(4): 275–276. doi: 10.1016/0039-9140(83)80062-9 18963357

[284] Morita K, Kaneko E. Spectrophotometric determination of arsenic in water samples based on micro particle formation of ethyl violet-molybdoarsenate. Anal Sci. 2006; 22(8):1085–1089. doi: 10.2116/analsci.22.1085 16896247

[285] Morita K, Kaneko E. Spectrophotometric determination of trace arsenic in water samples using a nanoparticle of ethyl violet with a molybdate−iodine tetrachloride complex as a probe for molybdoarsenate. Anal Chem. 2006; 78(22):7682–7688. doi: 10.1021/ac061074h 17105159

[286] Tan ZQ, Liu JF, Yin YG, Shi QT, Jing CY, Jiang GB. Colorimetric Au nanoparticle probe for speciation test of arsenite and arsenate inspired by selective interaction between phosphonium ionic liquid and arsenite. ACS Appl Mater Interfaces. 2014; 6(22):19833–19839. doi: 10.1021/am5052069 25335190

[287] Shan Y, Mehta P, Perera D, Varela Y. Cost and efficiency of arsenic removal from groundwater: A review. 2018. Available from: https://inweh.unu.edu/wp-content/uploads/2019/02/Cost-and-Efficiency-of-Arsenic-Removal-from-Groundwater-A-Review.pdf. Cited 20 June 2020.

[288] Bordoloi S, Nath SK, Gogoi S, Dutta RK. Arsenic and iron removal from groundwater by oxidation–coagulation at optimized pH: laboratory and field studies. J Hazard Mater. 2013; 260: 618–626. doi: 10.1016/j.jhazmat.2013.06.017 23827730

[289] Amrose SE, Bandaru SRS, Delaire C, van Genuchten CM, Dutta A, DebSarkar A, et al. Electro-chemical arsenic remediation: field trials in West Bengal. Sci Total Environ. 2014; 488: 539–546. doi: 10.1016/j.scitotenv.2013.11.074 24355249

[290] Mondal S, Roy A, Mukherjee R, Mondal M, Karmakar S, Chatterjee S, et al. A socio-economic study along with impact assessment for laterite based technology demonstration for arsenic mitigation. Sci Total Environ. 2017; 583: 142–152. doi: 10.1016/j.scitotenv.2017.01.042 28117153

[291] Brady NC., The nature and properties of soils. 9th ed. New York: Macmillan Publishing Company; 1984.

[292] Shan H, Ma T, Wang Y, Zhao J, Han H, Deng Y, et al. A cost-effective system for in-situ geological arsenic adsorption from groundwater. J Contam Hydrol. 2013; 154: 1–9. doi: 10.1016/j.jconhyd.2013.08.002 24035830

[293] Bohn HL, McNeal BL, O’Connor GA. Soil chemistry. New York: John Wiley & Sons; 1979.

[294] Bhattacharya P, Jacks G, Frisbie SH, Smith E, Naidu R, Sarkar B. Arsenic in the environment: A global perspective. In Sarkar B. (Ed.), Heavy metals in the environment. New York: Marcel Dekker; 2002, pp. 145–215.

[295] Sen Gupta B, Chatterjee S, Rott U, Kauffman H, Bandopadhyay A, DeGroot W, et al. A simple chemical free arsenic removal method for community water supply–A case study from West Bengal, India. Environ Pollut. 2009; 157(12): 3351–3353. doi: 10.1016/j.envpol.2009.09.014 19819054

[296] Skoog DA, Holler FJ, Crouch SR. Principles of instrumental analysis. 7th ed. Boston: Cengage Learning; 2018, pp. 231−246, 253−270.

[297] McLafferty FW. Interpretation of mass spectra. 3rd ed. Mill Valley, CA: University Science Books; 1980, pp. 1–44.

[298] Rosman KJR, Taylor PDP. Isotopic compositions of the elements 1997. Pure & Appl Chem. 1998; 70(1): 217−235. doi: 10.1351/pac199870010217

[299] Agilent Technologies, Inc. Comparing collision/reaction cell modes for the measurement of interfered analytes in complex matrices using the Agilent 7700 Series ICP-MS. Santa Clara, CA: Agilent Technologies, Inc.; 2009. Available from: https://www.agilent.com/cs/library/technicaloverviews/Public/5990-3236EN.pdf. Cited 21 August 2017.

[300] Chen ZL, Khan NI, Owens G, Naidua R. Elimination of chloride interference on arsenic speciation in ion chromatography inductively coupled mass spectrometry using an octopole collision/reaction system. Microchem J. 2007; 87(1): 87–90. doi: 10.1016/j.microc.2007.05.011

[301] Balcaen L, Bolea-Fernandez E, Resano M, Vanhaecke F. Inductively coupled plasma–Tandem mass spectrometry (ICP-MS/MS): A powerful and universal tool for the interference-free determination of (ultra)trace elements–A tutorial review. Anal Chim Acta. 2015; 894: 7–19. doi: 10.1016/j.aca.2015.08.053 26423624

[302] Darrouzès J, Bueno M, Lespès G, Holeman M, Potin-Gautie M. Optimisation of ICPMS collision/reaction cell conditions for the simultaneous removal of argon based interferences of arsenic and selenium in water samples. Talanta. 2007; 71(5): 2080–2084. doi: 10.1016/j.talanta.2006.09.019 19071567

[303] Long GL, Winefordner JD. Limit of detection. A closer look at the IUPAC definition. Anal Chem. 1983; 55(7): 712A–724A.

[304] Yan XP, Kerrich R, Hendry MJ. Determination of (ultra)trace amounts of arsenic(III) and arsenic(V) in water by inductively coupled plasma mass spectrometry coupled with flow injection on-line sorption preconcentration and separation in a knotted reactor. Anal Chem. 1998; 70(22): 4736–4742. doi: 10.1021/ac980654e 9844570

[305] Amaral CDB, Amais RS, Fialho LL, Schiavo D, Amorim T, Nogueira ARA, et al. A novel strategy to determine As, Cr, Hg and V in drinking water by ICP-MS/MS. Anal Methods. 2015; 7: 1215–1220.

[306] Bolea-Fernandez E, Balcaen L, Resano M, Vanhaecke F. Interference-free determination of ultra-trace concentrations of arsenic and selenium using methyl fluoride as a reaction gas in ICP–MS/MS. Anal Bioanal Chem. 2015; 407: 919–929. doi: 10.1007/s00216-014-8195-8 25260411

[307] Cutter LS, Cutter GA, San Diego-McGlone MLC. Simultaneous determination of inorganic arsenic and antimony species in natural waters using selective hydride generation with gas chromatography/photoionization detection. Anal Chem. 1991; 63(11): 1138–1142. doi: 10.1021/ac00011a015

[308] Ahmad A, Van der Wens P, Baken K, de Waal L, Bhattacharya P, Stuyfzand P. Arsenic reduction to <1 μg/L in Dutch drinking water. Environ Int. 2020; 134(105253). doi: 10.1016/j.envint.2019.105253 31810053

[309] Abejón A, Garea A, Irabien A. Arsenic removal from drinking water by reverse osmosis: Minimization of costs and energy consumption. Sep Purif Technol. 2015; 144: 46–53. doi: 10.1016/j.seppur.2015.02.017

[310] UN-Water. Compendium of water quality regulatory frameworks: which water for which use? New York: United Nations; 2015. Available from: https://www.unwater.org/app/uploads/2017/05/Compendium-of-Water-Quality-Main-Report_4.pdf. Cited 13 July, 2021.

[311] Ramsay L, Petersen MM, Hansen B, Schullehner J, van der Wens P, Voutchkova D, et al. Drinking water criteria for arsenic in high-income, low-dose countries: the effect of legislation on public health. Environ Sci Tech. 2021; 55: 3483–3493. doi: 10.1021/acs.est.0c03974 33635640

